# Combinatorial regulation by ERK1/2 and CK1δ protein kinases leads to HIF-1α association with microtubules and facilitates its symmetrical distribution during mitosis

**DOI:** 10.1007/s00018-024-05120-7

**Published:** 2024-02-01

**Authors:** Christina Arseni, Martina Samiotaki, George Panayotou, George Simos, Ilias Mylonis

**Affiliations:** 1 Laboratory of Biochemistry, Faculty of Medicine, University of Thessaly, 41500 Larissa, Greece; 2 Institute for Bio-Innovation, BSRC “Alexander Fleming”, 16672 Vari, Greece; 3 Gerald Bronfman Department of Oncology, Faculty of Medicine, McGill University, Montreal, Canada

**Keywords:** Hypoxia, HIF-1α, Casein kinase 1δ, ERK1/2, Microtubules, Mitosis

## Abstract

Hypoxia-inducible factor-1 (HIF-1) is the key transcriptional mediator of the cellular response to hypoxia and is also involved in cancer progression. Regulation of its oxygen-sensitive HIF-1α subunit involves post-translational modifications that control its stability, subcellular localization, and activity. We have previously reported that phosphorylation of the HIF-1α C-terminal domain by ERK1/2 promotes HIF-1α nuclear accumulation and stimulates HIF-1 activity while lack of this modification triggers HIF-1α nuclear export and its association with mitochondria. On the other hand, modification of the N-terminal domain of HIF-1α by CK1δ impairs HIF-1 activity by obstructing the formation of a HIF-1α/ARNT heterodimer. Investigation of these two antagonistic events by expressing double phospho-site mutants in *HIF1A*^−/−^ cells under hypoxia revealed independent and additive phosphorylation effects that can create a gradient of HIF-1α subcellular localization and transcriptional activity. Furthermore, modification by CK1δ caused mitochondrial release of the non-nuclear HIF-1α form and binding to microtubules via its N-terminal domain. In agreement, endogenous HIF-1α could be shown to co-localize with mitotic spindle microtubules and interact with tubulin, both of which were inhibited by CK1δ silencing or inhibition. Moreover, CK1δ expression was necessary for equal partitioning of mother cell-produced HIF-1α to the daughter cell nuclei at the end of mitosis. Overall, our results suggest that phosphorylation by CK1δ stimulates the association of non-nuclear HIF-1α with microtubules, which may serve as a means to establish a symmetric distribution of HIF-1α during cell division under low oxygen conditions.

**Supplementary Information:**

The online version contains supplementary material available at 10.1007/s00018-024-05120-7.

## Introduction

Oxygen deprivation or hypoxia can be established in cells and tissues under both physiological and pathological conditions, including cancer. Solid tumors have been shown to contain hypoxic areas attributable to enhanced cell proliferation rates and aberrant microcirculation [1]. Hypoxia Inducible Factor-1 (HIF-1) is the transcriptional activator of a wide range of biological processes that enable cells to adapt to and survive under hypoxia. HIF-1 is a heterodimer consisting of a constitutively expressed HIF-1β subunit (usually called ARNT; Aryl Hydrocarbon Receptor Nuclear Translocator) and an oxygen-regulated HIF-1α subunit that is frequently overexpressed in human cancers and has been connected to cancer cell survival and resistance to conventional anti-cancer treatments [2]. The machinery that controls the cellular levels of HIF-1α involves oxygen-sensing enzymes called PHDs (prolyl-hydroxylases), which, under physiological oxygen concentrations, hydroxylate HIF-1α and cause its association with an E3 ligase complex component (pVHL; Von Hippel–Lindau) which promotes its degradation to the proteasome [3]. As oxygen levels drop, PHDs are inactivated, leading to HIF-1α stabilization, the formation of a functional HIF-1 complex inside the nucleus, and HIF-1 binding to specific DNA sequences called hypoxia response elements (HREs) [4]. In addition to this elegant regulation, HIF-1 activation may also involve several additional steps, including increased transcription of the *HIF1A* gene by NF-κB and STAT3, enhanced translation of its mRNA by activation of the PI-3K/AKT pathway [5–7], and post-translational modification, mainly phosphorylation mediated by several different protein kinases [8]. Modification of the N-TAD domain of HIF-1α by glycogen synthase kinase 3 destabilizes HIF-1α in a VHL-independent manner [9, 10]. On the opposite, phosphorylation by ATM or PKA stimulates HIF-1α by stabilizing its protein levels or enhancing its interaction with transcriptional cofactors CBP/p300 [11, 12]. Moreover, cell cycle-regulated protein kinases CDK1 and CDK2 have been shown to target HIF-1α and either promote or inhibit its stabilization, respectively [13, 14]. We have previously identified two distinct phosphorylation events mediated by CK1δ and ERK1/2 protein kinases [15, 16] that do not affect HIF-1α stability but rather negatively or positively, respectively, affect HIF-1 activity by controlling the interaction of HIF-1α with other proteins. CK1δ targets Ser247 in the PASB domain of HIF-1α and inhibits HIF-1 activity by impairing the formation of a functional HIF-1α/ARNT heterodimer [15, 17]. Instead, ERK1/2-mediated phosphorylation of Ser641 and/or Ser643 within the ETD (ERK1/2-targeted domain) of HIF-1α stimulates HIF-1 activity in two ways: first, by blocking its interaction with the major exportin CRM1 ensuring its nuclear accumulation and second, by enhancing its binding to the histone chaperone NPM1 which is essential for HIF-1-mediated transcriptional activation [18, 19]. Blocking ERK1/2-mediated phosphorylation of HIF-1α by kinase inhibitors, serum starvation or mutations of the ERK1/2 sites drives HIF-1α outside the nucleus and onto the outer mitochondrial membrane via its association with mortalin. Formation of this complex, which also includes hexokinase II (HKII) and the voltage-dependent anion channel (VDAC1), impairs the translocation of BAX (BCL2-associated X) onto the mitochondrial membrane under stress conditions and protects from apoptosis [20, 21]. It is, therefore, possible that HIF-1α can also exert non-genomic cytoplasmic functions that can support cell survival under low oxygen even in the absence of, before, or even simultaneously with hypoxia-inducible gene expression. In support of such a notion, additional non-transcriptional nuclear or cytoplasmic functions of HIF-1α have also been described, such as its involvement in cell-cycle arrest or the activation of Notch pathway via its interaction with cdc6 or γ-secretase, respectively [22, 23]. These findings suggest the existence of two distinct subcellular HIF-1α pools: a nuclear HIF-1α fraction engaged in transcription in complex with ARNT and a non-nuclear fraction of HIF-1α engaged as a monomer in non-genomic functions. The balance between these two separate HIF-1α pools may depend on its nucleocytoplasmic trafficking and its anchorage on different cellular structures via association with different protein partners [20, 24, 25], a subject which is largely unexplored.

Microtubules, in addition to their role in cellular motility and chromosome segregation, are major determinants of intracellular trafficking of proteins, vesicles, and organelles [26]. Microtubule dynamics and turnover significantly increase as the cell cycle progresses from interphase to mitosis. The tubulin heterodimer as well as MAPs (microtubule-associated proteins) are decorated by many post-translational modifications. The phosphorylation status of MAPs has been associated with shaping microtubule dynamics [27, 28]. For example, MAP1B is a target for phosphorylation by several different kinases such as Glycogen synthase kinase IIIβ and DYRK1A (Dual-specificity tyrosine-phosphorylation-regulated kinase) affecting microtubule stability [29]. During mitosis, the mitotic spindle, a bipolar assembly of microtubules, uses microtubule-based proteins to segregate sister chromatids. This essential cellular process must be strictly regulated involving various phosphorylation events. More specifically, the components of the mitotic kinome include several kinase families such as NIMA-related kinases (Neks), cyclin-dependent kinases (CDKs), Polo-like kinases (Plks), and Aurora kinases, as well as phosphatases. Cell cycle alters the regulation of more than 1000 phosphoproteins, and site-specific phosphorylation activates or downregulates mitosis [30].

Additionally, kinases such as c-Jun N-terminal kinases (JNK) and p38 kinases, as well as the casein kinase 1 (CK1) family of protein kinases are important mediators of response to stress due to DNA damage, inflammation, or metabolic stress. Physiologically, this kinase family that consists of seven CK1 isoforms (α, β, γ1, γ2, γ3, δ, and ε) in mammals is responsible for the regulation of cell cycle, differentiation, and apoptosis [31]. CK1 family members have been shown to target dynein and drive the transport of membrane organelles along microtubules [32]. Moreover, CK1 isoforms have been associated with microtubule dynamics and chromosome segregation as CK1α, δ and ε are associated with microtubules and centrosomes by anchoring to scaffold proteins such as AKAP450 to exert their function [33]. Furthermore, CK1δ, associates with the spindle apparatus and directly modulates microtubules by phosphorylation of α-, β, and γ- tubulin, thereby exerting stress-induced functions at the spindle apparatus and the centrosome [34]. Moreover, it can phosphorylate tubulin, MAPs and Tau affecting microtubule and genomic stability [33]. Notably, several stress- and cancer-related transcription factors, such as NF-κB, p53, Androgen Receptor, and HIF-1α, are known to associate with microtubule proteins in interphase cells, which may facilitate their transport to the nucleus [35–38]. Intriguingly, both microtubule-stabilizing and destabilizing agents could impair HIF-1α nuclear accumulation and activity [39].

To investigate the interplay between the two aforementioned and antagonistic HIF-1α phosphorylations by CK1δ and ERK1/2 and its role in the subcellular distribution and activities of HIF-1α, we constructed a number of HIF-1α mutant forms that combined phosphodeficient and phosphomimetic mutations in both sites of these two kinases and expressed them in a *HIF1A* knockout cell line. Moreover, we investigated the interactome of non-nuclear HIF-1α and how this may be affected by CK1δ. We show that modification of non-nuclear HIF-1α by CK1δ causes its release from the mitochondria and its association with microtubules via an interaction between the N-terminal part of HIF-1α and tubulin. Furthermore, our results show that the CK1δ-stimulated association of endogenous HIF-1α with microtubules is most prominent during mitosis and is required for the symmetrical delivery of HIF-1α to the daughter nuclei during cell division.

## Materials and methods

### Plasmids

Cloning of HIF-1α 1–347 or its S247A (Serine 247 to Alanine; Ser247Ala) and S247D (Serine 247 to Aspartate; Ser247Asp) mutant forms into the mammalian expression vector pFLAG-CMV2 (Sigma-Aldrich, St Louis, MO, USA) or the bacterial expression vector pGEX-4T1 (Amersham Biosciences, Little Chalfont, UK) was performed with PCR by using the respective previously reported full-length pGEX-4T1-HIF-1α plasmids and a suitable set of primers [15] (Table S1). Full-length GFP-HIF-1α WT and single GFP-HIF-1α S641/643A (Serines 641 and 643 to Alanines; Ser641/643Ala), GFP-HIF-1α S641E (Serine 641 to Alanine; Ser641Glu), GFP-HIF-1α S247D, and S247A mutant forms were previously described [15, 16]. Cloning of HIF-1α fragments 348–826 or 575–826 into the bacterial expression vector pGEX-4T1 (Amersham Biosciences, Little Chalfont, UK) was previously described [16].

To create HIF-1α mutant forms combining mutations in CK1δ-site with the mutations in ERK1/2-site, we used the previously described pEGFP-C1-HIF-1α S641/643A or S641E plasmids [16, 19] as a template for site-directed mutagenesis of Ser 247 by using QuikChange II mutagenesis kit (Agilent, Santa Clara CA, USA) and a suitable set of primers (Tables S1, S2). Plasmids expressing HIF-1α mutant forms were verified by sequencing (CeMIA, Larissa, Greece).

### Cell culture and transfection

Human cervical carcinoma HeLa_S3 cells (CVCL_0058; ATCC) and human breast cancer MCF‐7 cells (CVCL_0031; a gift from Dr. P. Moutsatsou (Medical School, University of Athens, Greece; originally acquired from ATCC) were regularly tested for mycoplasma and cultured in Dulbecco's modified Eagle's medium (DMEM; Biosera, Nuaille, France) supplemented with 10% FBS (Biosera Nuaille, France) and 100 U/mL penicillin–streptomycin (Biochrom, Berlin, Germany). For hypoxic treatment, cells were exposed for 4–24 h to 1% O_2_, 94% N_2,_ and 5% CO_2_ in an IN VIVO2 200 hypoxia workstation (Baker Ruskinn, Sanford, ME). When required, cells were treated for 4–16 h with the CK1 inhibitors D4476 (10 μΜ) or IC261 (2 μΜ), nocodazole (40 ng/mL), paclitaxel (10 nM) or RO-3306 (5 μΜ) (Table S3). Cells were treated with 40 ng/mL of nocodazole for 6 h to be synchronized. Then, cells were washed and supplemented with fresh growth medium for 1 h to release. Alternatively, 5 μΜ of RO-3306 was used to arrest cells at G2 and then, cells were observed after their release with fresh medium for 0 to 90 min. Transient transfections with plasmid DNA were carried out using the JetPRIME® Polyplus or the TurboFect transfection reagents (Table S2) according to the manufacturer’s instructions. For CK1δ siRNA-mediated silencing, cells were incubated for 24 h with the indicated siRNAs in the presence of VIROMER®BLUE reagent (Tables S2, S4).

### HeLa *HIF1A*^−/−^ cell line construction by CRISPR/Cas9

**Fig. 1:**
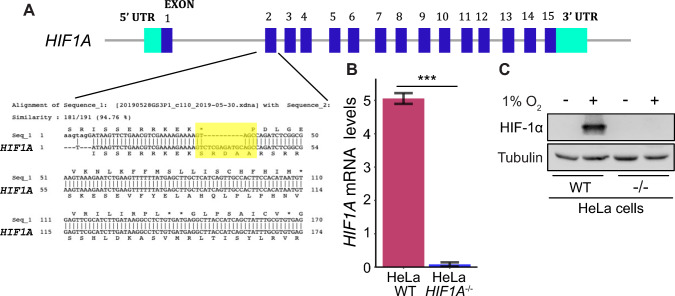
Construction of a *HIF1A*^−/−^ knock out HeLa-derived cell line by CRISPR/Cas9.** A** Depiction of *HIF1A* gene and its alignment with the sequence (Seq_1) derived from DNA sequencing results of a HeLa-derived cell line after CRISPR/Cas9 editing. Yellow highlight shows the indel region. **B** *HIF1A* mRNA levels in HeLa WT or *HIF1A*^−/−^ cells under hypoxia (1% O_2_) as determined by RT-PCR (as indicated). Results are shown as ratios in relation to HeLa WT and represent the mean of two independent experiments performed in triplicates ± s.d. (*n* = 6; ***p < 0.001). **C** Western Blot analysis of total cell extracts from HeLa WT or *HIF1A*^−/−^ cells grown in normoxia or hypoxia (1% O_2_) using antibodies against the indicated proteins. Tubulin was used as loading control

To construct a *HIF1A*^−/−^ HeLa cell line not expressing endogenous HIF-1α, we used the *HIF1Α* CRISPR/Cas9 Double Nickase system (Table S2) and the parental HeLa_S3 cell line (CVCL_0058; ATCC) according to manufacturers’ instructions. Briefly, the sgRNAs, HIF-1α-sgRNA-puro, and HIF-1α-sgRNA-GFP plasmids were transfected into HeLa cells (2.5 × 10^5^ cells) with the JetPRIME® Polyplus Transfection reagent (Table S2). After 48 h, the medium was replaced with fresh medium containing puromycin (Gibco, USA) at a concentration of 2.5 μg/ml to select colonies. All viable cell colonies were tested by Western Blotting for protein expression and real-time PCR for *HIF1A* mRNA expression levels. Colonies with no detectable HIF-1α signal under hypoxia were further verified for *HIF1A* knockout by sequencing (CeMIA, Larissa, Greece; Fig. 1).

### RNA extraction, Real-time PCR, and Reporter gene assay

HeLa cells were transfected with plasmids expressing different GFP-HIF-1α mutant forms. 24 h post-transfection cells were washed with PBS, and total RNA from transfected HeLa cells was isolated using the Nucleozol reagent (Macherey–Nagel, Duren, Germany). cDNA was synthesized with the High-Capacity cDNA Reverse Transcription synthesis kit (Thermo Fisher Scientific, Waltham, MA, USA). Real-time PCR was performed with SYBR™ Select Master Mix (Thermo Fisher Scientific, Waltham, MA, USA) in a LightCycler® 96 System (Roche, Basel, Switzerland). The mRNAs encoding *P4HA1, HIF1A, or ACTB* were amplified using primers reported in Table S1. Each sample resulting from 3 biological replicates was assayed in triplicate for both target and internal control. Relative quantitative gene expression was calculated by using the ΔΔCT method.

Reporter gene assays were performed as previously described by using pGL3-5xHRE-VEGF-*Luciferase* and pCI-*Renilla* reporter plasmids [16], and luciferase activity was determined by using the ‘Dual-Luciferase Reporter Assay System’ (Table S2) measured in a Spark® multimode microplate reader (Tecan Trading AG, Switzerland).

### Protein purification and Western Blotting

GST-tagged mutant HIF-1α forms and their fragments were expressed in *Escherichia coli* and purified as previously reported for GST–HIF-1α [40]. Protein analysis by SDS-PAGE was followed by Western Blotting using specific antibodies (Table S5). Western Blotting images were taken using a Uvitec Cambridge Chemiluminescence Imaging System (Uvitec Cambridge, Cambridge, UK) using Alliance software, version 16.06, and quantified by Uviband Software (Uvitec Cambridge, Cambridge, UK).

### In vitro binding assays and phosphorylation

Approximately 10 μg of purified GST, GST-tagged HIF-1α 348–826, 575–826, 1–347 or HIF-1α 1–347 mutant forms were immobilized on 25 μl glutathione-Sepharose beads and incubated with protein extracts (∼1 mg of total protein) from HeLa cells lysed with PME buffer (0.1 M MOPS, 2 mM EGTA, 1 mM MgSO_4_, 1 mM DTT, 0.1 mM GTP, 1 mM PMSF) for 4 h at 4 °C [41]. Precipitates were washed with PBS-1%Tween-20 and 1 mM PMSF. Bound proteins were eluted by 2 × SDS-PAGE loading buffer and analyzed by SDS-PAGE and Western Blotting (Table S5). When required, in vitro phosphorylation reactions were carried out as previously described [16] by using 100 units of recombinant active CK1δ (New England Biolabs, USA) on bound GST-HIF-1α or its truncated forms and 1 mM ATP for 1 h at 30 °C before the incubation with the cellular extracts.

### Biochemical fractionation and microtubule stabilization assay

To obtain crude biochemical fractions of endogenous or overexpressed HIF-1α, cells were washed with prewarmed PBS (37 °C) and lysed with prewarmed MTS-buffer (0.1 M MOPS, 2 mM EGTA, 5 mM MgCl_2_, 2 M glycerol, 2.5 mM GTP, 0.1% NP-40, 1 mM PMSF) followed by incubation at 37 °C for 15 min with gentle shaking every 5 min to permeabilize the cells according to Sato et al. [42]. The permeabilized cells were collected and centrifuged (16.000×*g*, 1 h, 37 °C). The resulting supernatant (soluble fraction) was mixed with 2 × SDS sample buffer supplemented with 5% DTT. The pellet that corresponds to the cytoskeletal fraction was resuspended in 2 × SDS sample buffer to collect a fraction enriched in microtubules and microtubule-associated proteins.

To isolate a fraction containing stabilized microtubules, we followed the protocol reported by Sloboda et al. [41]. Briefly, HeLa *HIF1A*^−/−^ cells were seeded and transfected with pFLAG-CMV2-HIF-1α 1–347 or its point mutants, washed with ice-cold PBS, lysed with 1 ml of PME buffer and homogenized by using an insulin syringe and a mechanical homogenizer. The lysates were incubated on ice for 10 min and were centrifuged at 17.000 × *g* for 60 min at 4 °C. Then, 20 μΜ of paclitaxel was added to the supernatant and incubated for 20 min at 37 °C. After incubation, a PME-10% sucrose pillow was added to tubes, and the supernatant was added carefully above the sucrose pillow. Ultracentrifugation was performed at 45,000 × *g* for 30 min at 20 °C in a Sorvall PRO ultraspeed centrifuge. The pellet was resuspended in PME buffer containing 10 μΜ paclitaxel. The ultracentrifugation step was repeated to collect in the pellet the isolated microtubules and their associated proteins, which was then resuspended in PME- paclitaxel (10 μM) buffer and processed for SDS-PAGE electrophoresis.

### Measurement of Caspase 3/7 activity

HeLa *HIF1A*^−/−^ cells were transfected with GFP, GFP-HIF-1α S641/3A and GFP-HIF-1α SDSA. At 24 h post-transfection, cells were treated either with 75 μM etoposide or with DMSO as solvent control for 4 h under normoxia. Caspase 3 and 7 activity was measured as previously described [20] with the ‘Caspase-Glo® 3/7 Assay’ kit (Promega, Madison, WI, USA) by using the Spark® multimode microplate reader (Tecan Trading AG, Switzerland).

### Immunoprecipitation and mass spectrometry analysis

Immunoprecipitation (IP) experiments were performed as previously described [19]. Briefly, HeLa cell total extracts or transfected HeLa *HIF1A*^−/−^ lysates were incubated for 16 h at 4 °C in HNMT buffer (25 mM Hepes pH 7.5, 150 mM NaCl, 2 mM MgCl_2_, 1% Triton X-100, 0.5 mM dithiothreitol and 1 mM PMSF) with protein A or G beads supplemented with 1 μg of anti-HIF-1α, anti-GFP or anti-tubulin antibodies (Table S5). Precipitates were eluted with SDS loading buffer and analyzed by SDS-PAGE, immunoblotting, or silver nitrate dye for mass spectrometry analysis.

The protein bands that had been stained by silver nitrate were destained and processed according to the classical in-gel tryptic digestion protocol [20, 43]. The peptide samples were analyzed on a liquid chromatography-tandem mass spectrometry (LC–MS/MS) setup consisting of a Dionex Ultimate 3000 nanoRSLC coupled in line with a Thermo Q Exactive HF-X Orbitrap mass spectrometer operating in a Top10 Data Dependent Acquisition mode in the scan range of 350–1500 m/z using 120 K resolving power with an AGC of 3 × 106 and max IT of 100 ms, followed by MS/MS scans of the 10 most abundant ions, using 15 K resolving power with an AGC of 1 × 105 and max IT of 100 ms and an NCE of 28. The peptide sample was run using a preconcentration setup using an Acclaim PepMap precolumn and a 50 cm long column (Thermo Scientific™) operating in an oven of 55 °C. The total analysis time for the sample was 60 min.

The Rawfiles generated were processed using the MaxQuant 1.6.17.0 to identify and quantify the proteins present in the HIF-1α IP samples, according to the LFQ algorithm. The runs were analyzed with the Andromeda search engine against the reviewed UniProt proteome downloaded from the UniProtKB database. The precursor and the fragment mass tolerance allowed were set to 4.5 and 20 ppm, respectively. The minimum peptide length was set to seven amino acids, and trypsin was selected as the proteolytic enzyme, allowing up to two missed cleavage sites. Carbamidomethylation (Cys) was set as a fixed modification, while oxidation (Met), deamidation of (Asn) and (Gln), and N-term acetylation were the variable modifications. The false discovery rate (FDR) at both the protein and peptide levels was set to 1%. Proteins identified as common contaminants or binders of both GFP-HIF-1α wt-SA and GFP-HIF-1α SD-SA were filtered-out. Additionally, the identified proteins were assessed for their presence in the CRAPome database (v.2.0 https://reprint-apms.org/?q=chooseworkflow) [44] and processed as contaminants. The remaining lists of HIF-1α SD-SA and wt-SA interacting proteins were compared, and Gene Ontology for Cellular Components was performed with Gene Codis 4.0 [45] and R Studio (Version 2023.06.1 + 524; Posit Software, PBC).

### Immunofluorescence

Immunofluorescence microscopy experiments were performed as previously described (Mylonis 2017). In brief, cells were grown on coverslips and fixed with 3.7% formaldehyde in PBS for 10 min, permeabilized with 0.1% Triton-X 100 at 4 °C for 15 min and treated with 1% BSA in PBS for 1 h at room temperature. Then, coverslips were incubated overnight at 4 °C with specific antibodies (Table S3), washed twice with PBS, and incubated for 1 h at room temperature with appropriate secondary antibodies (Table S3). Cells were counterstained with DAPI (4′,6-diamidine-2′-phenylindole dihydrochloride; Sigma Aldrich, St Louis, MO, USA), mounted on slides, and visualized.

All images were acquired by a Zeiss Axio Imager.Z2 microscope equipped with an AxioCam MRm camera and a 40 × /0.75 objective or a 100 × /1.3 oil-immersion lens via Zeiss Immersol 518F. Available Zeiss filter sets used in this study were Fs02 (G365/FT510/LP420), Fs09/AF488 (BP450-490/FT510/LP515), Fs14/AF546 (BP510-560/FT580/LP590), and Fs26/AF660 (BP575-625/FT645/BP 660–710). Images collection was achieved by Zeiss Zen 2011 (blue edition, ver. 1.0.1.0) image acquisition software. For quantification purposes, cells were captured with the same exposure time at 22 °C (room temperature) and saved at 880 × 684 pixels (90.10 × 70.03 microns) in tagged image file format for downstream analysis. Image quantification was performed on unmodified images with ImageJ (NIH, Bethesda, MD, USA) software and plugins. For figure representation, images were enhanced, split, or cut using ImageJ (NIH, Bethesda, MD, USA) to accurately reflect the relationships between factors quantified from unmodified images.

### Statistical analysis

Statistical differences between the two groups of data were evaluated using the GraphPad Prism version 5.04 software or R software (version 4.2.2; The R Foundation for Statistical Computing) with RStudio (Version 2023.06.1 + 524; Posit Software, PBC). Differences were evaluated by Student’s *t*-test (two-tailed) between two groups or by one-way analysis of variance (ANOVA) within multiple groups; *P* < 0.05 was deemed as statistically significant.

## Results

### Analysis of double phosphosite mutants of HIF-1α reveals its combinatorial control by CK1δ and ERK1/2

As outlined above, HIF-1α lacking the ERK1/2-mediated modification resides outside the nucleus and is inactive in terms of transcriptional regulation but may be active in terms of mediating non-nuclear functions such as regulation of apoptosis or Notch signaling. To explore the role of CK1δ in the function of non-nuclear HIF-1α, we wanted to analyze non-nuclear HIF-1α forms carrying mutations in the CK1δ site in cells lacking endogenous HIF-1α and grown under hypoxic conditions. To make this possible, we first constructed a *HIF1A* knockout HeLa cell line by using CRISPR/Cas9 technology. A parental HeLa_S3 cell line was transfected with the *HIF1Α* CRISPR/Cas9 Double Nickase system (Table S2). After colony selection with puromycin, viable clones were chosen, grown, sequenced, and assayed for the expression of endogenous HIF-1α (Fig. 1). There was at least one HeLa clone (referred to as HeLa *HIF1A*^−/−^) in which the *HIF1A* gene was disrupted in an area encompassing exon2 (Fig. 1A) and which expressed neither HIF-1α mRNA nor HIF-1α protein under hypoxia (1% Ο_2_; Fig. 1B, C).

**Fig. 2:**
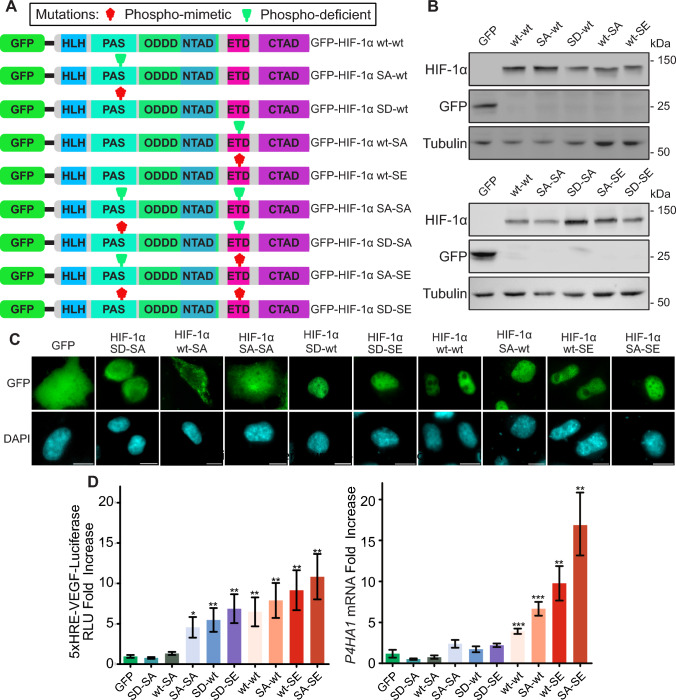
Analysis of double phosphosite mutants of HIF-1α reveals its combinatorial control by CK1δ and ERK1/2. **A** Schematic representation of GFP-HIF-1α forms depicting mutations on sites modified by CK1δ and ERK1/2 (as indicated). **B** Western Blot analysis of GFP-tagged HIF-1α phosphorylation mutant forms (as indicated) expressed in HeLa *HIF1A*^*−/−*^ cells under hypoxia (1% O_2_) using antibodies against HIF-1α (top panel) or GFP (middle panel). Tubulin was used as loading control (bottom panel). Blots are representative of three independent experiments. **C** Fluorescence microscopy images of GFP, GFP-HIF-1α WT, and its mutant forms as indicated and depicted in **A** in HeLa *HIF1A*^*−/−*^ cells grown under hypoxia (1% O_2_). Nuclei were stained with DAPI (Scale bars: 10 μm). **D** *P4HA1* mRNA (right panel) or Relative Luciferase Units (RLU; left panel) fold increase levels in HeLa *HIF1A*^*−/−*^ cells under hypoxia (1% O_2_) expressing GFP or GFP-HIF-1α forms as determined by RT-PCR or reporter gene assays (as indicated). Results are shown as ratios in relation to cells expressing GFP alone and represent the mean of three independent experiments performed in triplicates ± SEM (*n* = 9; *p < 0.05; **p < 0.01)

This *HIF1A*^−/−^ cell clone was then used to transiently express the GFP-tagged HIF-1α mutant variants that combined mutations in the sites targeted by CK1δ and ERK1/2. More specifically, the CK1δ site phosphodeficient mutant carrying the Ser247Ala mutation (or SA-wt) and the phosphomimetic mutant with the Ser247Asp mutation (or SD-wt) were combined with the constitutively non-nuclear HIF-1α form that lacks the ERK1/2 sites, i.e. GFP-HIF-1α S641/643A carrying Ser641Ala and Ser643Ala mutations (or wt-SA). For control purposes, the same CK1δ site single mutants were combined with the constitutively nuclear HIF-1α that harbors a phospho-mimetic mutation in the ERK1/2 sites, i.e. GFP-HIF-1α S641E carrying the Ser641Glu mutation (or wt-SE). Thus, we created mutant forms that carried phosphodeficient and/or phosphomimetic mutations in both kinase sites (depicted as SA-SA, SD-SA, SA-SE and SD-SE and shown schematically in Fig. 2A).”

These single or double phosphosite HIF-1α mutant forms were expressed at levels without statistically significant differences under hypoxia (1% Ο_2_) in the *HIF1A*^−/−^ cells (Fig. 2B, Sup. Fig. S1A–D). However, they exhibited a graded subcellular localization. More specifically, there was perinuclear staining for the Ser247Asp-Ser641Asp/S641/643A (SD-SA) mutant, nuclear exclusion for the S641/643A (wt-SA) mutant and whole cell staining for the Ser247Ala-S641/643A (SA-SA) mutant. On the other hand, the wild-type (wt-wt) form as well as the single or double phosphosite mutants SD-wt, SA-wt, wt-SE, SA-SE and SD-SE we exclusively nuclear (Fig. 2C). These results are in agreement with our published data with the single phosphosite mutants, which were previously analyzed in normoxic *HIF1A*^+/+^ cells [15, 16, 19]. Furthermore, the results with the SA-SA mutant revealed that complete lack of the CK1δ phosphorylation at Ser247 partially restores the mislocalization defect of the S641/643A mutation, probably because the increased affinity for nuclear ARNT (due to the S247A mutation) and the subsequent nuclear retention counterbalances the active CRM1-dependent nuclear export triggered by the S641/643A mutation and lack of ERK1/2-mediated phosphorylation.

The localization results correlated tightly with the results of luciferase-based HIF-1 transcriptional activity and real-time PCR-based HIF-1 target gene expression measurements of the same cells under hypoxia (Fig. 2D). More specifically, the mostly non-nuclear HIF-1α double and single phosphosite mutants (namely SD-SA and wt-SA, respectively) were equally inactive, as both lack ERK1/2 phosphorylation, while the single phosphosite HIF-1α mutants that were previously shown to be nuclear and possess higher affinity for ARNT (SA-wt; [15]) or for NPM1 and chromatin components (wt-SE; [18]) exhibited, as expected, higher transcriptional activity than the wild-type form. Interestingly, the lack of the CK1δ-mediated modification combined with the phosphomimetic mutation at the ERK1/2 site (in the SA-SE mutant) created an even more active HIF-1α form suggesting independent and additive effects of the modifications catalyzed by the two kinases. In the same vein, the phosphodeficient mutation at the CK1δ site can partly complement the inactivating effect of the phosphodeficient mutation in the ERK1/2 site as shown by the activity of the SA-SA mutant (as also discussed above). Taken together, these results show that HIF-1α is partially modified by both kinases in HeLa cells, and the measurable activity of the wild-type form is the result of the balance between the two opposing phosphorylation events. Furthermore, they support the idea that the interplay between these two modifications can create a gradient of subcellular localization and transcriptional activity HIF-1α that can serve and fine-tune both the nuclear/transcriptional and the non-nuclear/non-transcriptional roles played by possibly two functionally distinct HIF-1α pools.

### The CK1δ phosphomimetic mutation reduces the association of non-nuclear HIF-1α with mitochondria

**Fig. 3:**
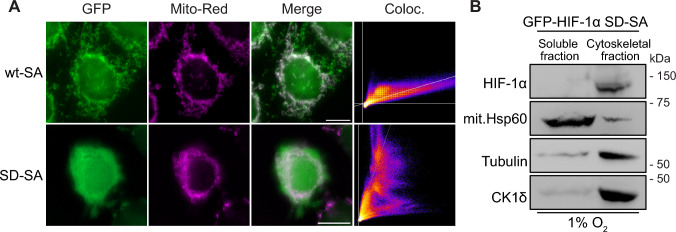
The CK1δ phosphomimetic mutation reduces the mitochondrial association of non-nuclear HIF-1α. **A** Fluorescence microscopy images of HeLa *HIF1A*^−/−^ cells grown under hypoxia (1% O_2_) expressing either GFP-HIF-1α wt-SA or GFP-HIF-1α SD-SA (as indicated). Cells were treated with Mito Red (100 nM) for 15 min prior to fixation for mitochondrial staining. Nuclei were stained with DAPI (Scale bars: 10 μm). Scatterplots depict colocalization analysis of representative images. **B** Western Blot analysis of proteins recovered in soluble and microtubule-rich fractions (as indicated) of HeLa *HIF1A*^−/−^ cells under hypoxia (1% O_2_) expressing GFP-HIF-1α SD-SA, using antibodies against the indicated proteins. Images are representative of two independent experiments

Examining more carefully the mutant localization results, we noticed that the non-nuclear SD-SA form, which is completely transcriptionally inactive like the wt-SA form, lacked the distinctive mitochondrial localization pattern of the wt-SA form (Fig. 2C). To further study this, we analyzed the co-localization of both these forms with mitochondria. As shown in Fig. 3A, the mitochondrial co-localization of the SD-SA form was significantly reduced compared to the wt-SA form, which was largely mitochondrial. At the same time, the SD-SA form did not become diffuse but appeared associated with cytoskeletal structures. To confirm these microscopic results biochemically, we performed crude fractionation of hypoxic HeLa *HIF1A*^−/−^ cells expressing GFP-HIF-1α SD-SA using a protocol that separates soluble and organelle proteins from cytoskeletal elements, mostly microtubule and actin filaments. As shown in Fig. 3B, the HIF-1α SD-SA form was recovered in the cytoskeletal fraction together with tubulin and CK1δ while the mitochondrial marker Hsp60 was largely recovered in the soluble fraction. Taken together, these results suggest that CK1δ-mediated phosphorylation releases non-nuclear HIF-1α from the mitochondrial surface and, at the same time, mediates its association with the cytoskeleton.

### The CK1δ phosphomimetic mutation stimulates the association of non-nuclear HIF-1α with tubulin

**Fig. 4:**
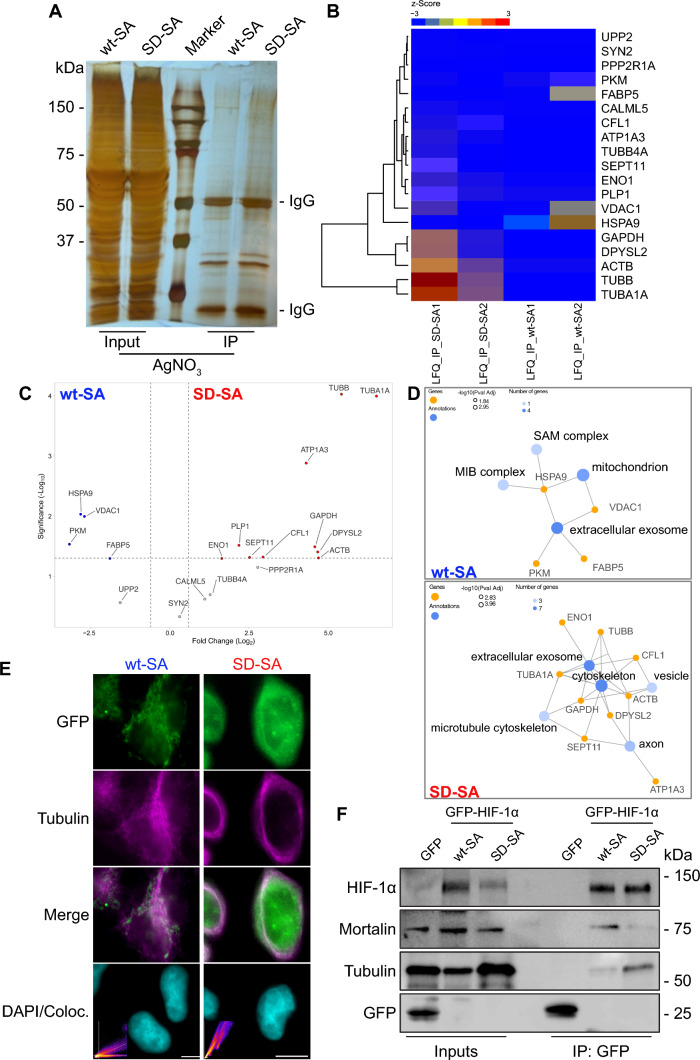
Proteomic analysis of proteins immunoprecipitated with cytoplasmic GFP-HIF-1α wt-SA or GFP-HIF-1α SD-SA forms reveals increased association of phospho-mimetic (CK1δ site) HIF-1α with microtubule proteins. **A** AgNO_3_ staining of total proteins (Input) and of GFP-immunoprecipitated proteins (IP) from HeLa *HIF1A*^−/−^ cells in hypoxia (1% O_2_) expressing either GFP-HIF-1α wt-SA or GFP-HIF-1α SD-SA (as indicated). Stained proteins were analyzed by mass spectrometry. **B** Heatmap of log2 LFQ values (Label-Free Quantification) of proteins immunoprecipitated by anti-GFP from HeLa *HIF1A*^−/−^ cells expressing either GFP-HIF-1α wt-SA or GFP-HIF-1α SD-SA (as indicated). Both biological replicates are represented (1 or 2; as indicated). **C** Volcano plot of immunoprecipitated proteins showing significantly altered association with GFP-HIF-1α wt-SA (Blue dots) or GFP-HIF-1α SD-SA (Red dots; as indicated) after mass spectrometry. Graph portrays the difference between the two samples expressed as log2(x) versus their statistical significance expressed as –Log10(*p*-value) from two biological replicates (*p* < 0.05). **D** Dot plot of GO Cellular Component ontology analysis of proteins that alter their association with GFP-HIF-1α wt-SA (upper panel) or GFP-HIF-1α SD-SA (lower panel). **E** Immunofluorescence microscopy images of HeLa *HIF1A*^−/−^ cells grown under hypoxia (1% O_2_) expressing either GFP-HIF-1α wt-SA or GFP-HIF-1α SD-SA (as indicated) using an antibody against tubulin to visualize microtubules. Nuclei were stained with DAPI (Scale bars: 10 μm). Insets depict scatterplots of colocalization analysis in the representative images. **F** Protein extracts (Input) or anti-GFP immunoprecipitated proteins (IP) of hypoxic (1% O_2_) HeLa *HIF1A*^−/−^ cells expressing GFP or GFP-HIF-1α forms (as indicated) were analyzed using antibodies against the indicated proteins. Images are representative of two independent experiments

To further explore our previous observation and identify possible CK1δ-dependent interactions of non-nuclear HIF-1α, GFP-tagged HIF-1α wt-SA and SD-SA forms were immunoprecipitated from total protein extracts of HeLa *HIF1A*^−/−^ cells grown under hypoxia (1% Ο_2_) and the co-immunoprecipitated proteins were analyzed by SDS-PAGE, excised from the gel matrix and identified by mass spectrometric analysis (Fig. 4A, Sup_File_S1). The interactomes of the two mutant forms varied significantly (Fig. 4B, C; Heatmap and volcano plot comparing the GFP-HIF-1α wt-SA and SD-SA interactomes; *p* < 0.05). GO cellular component enrichment analysis as well as clustering of the co-precipitated proteins showed that HIF-1α wt-SA associated mainly with mitochondrial proteins (Fig. 4D upper panel) while HIF-1α SD-SA was associated primarily with microtubular and cytoskeletal proteins (Fig. 4D lower panel). These proteomic results were verified by immunofluorescence microscopy and immunoprecipitation experiments in hypoxic HeLa *HIF1A*^−/−^ cells. The co-localization of the GFP-HIF-1α SD-SA form with tubulin was significantly stronger compared to the GFP-HIF-1α wt-SA form (Fig. 4E). Similarly, the association of immunoprecipitated GFP-HIF-1α SD-SA with tubulin was stronger than GFP-HIF-1α wt-SA and, conversely, the association of GFP-HIF-1α SD-SA with mortalin (the protein that targets HIF-1α to mitochondria) was weakened compared to GFP-HIF-1α wt-SA (Fig. 4F).

The mitochondrial fraction of endogenous HIF-1α or the HIF-1α wt-SA mutant form was previously shown to confer resistance to apoptosis under stress conditions [20, 21]. We, therefore, also analyzed the anti-apoptotic activity of the HIF-1α SD-SA form in comparison with the HIF-1α wt-SA form either in etoposide-treated HeLa *HIF1A*^−/−^ or in MCF7 cells under normoxia to also control for interference by endogenous HIF-1. Despite its weaker association with mitochondria and mortalin, HIF-1α SD-SA was still able to confer resistance to etoposide-induced apoptosis in both cell lines, a bit less efficiently but without statistically significant difference from HIF-1α wt-SA (Sup. Fig. S1E, F). This anti-apoptotic activity of the SD-SA mutant could be attributed to its residual presence on the mitochondria, its dynamic exchange between mitochondria and microtubules, or the physical and functional association between mitochondria and microtubules [46], possibly bridged by a HIF-1α and mortalin containing complex [20]. Overall, our results suggest that the level of association of non-nuclear HIF-1α with either microtubule or mitochondrial proteins depends on its phosphorylation by CK1δ.

### CK1δ-mediated phosphorylation of the HIF-1α N-terminal domain drives its association with tubulin and mitotic spindle microtubules

**Fig. 5:**
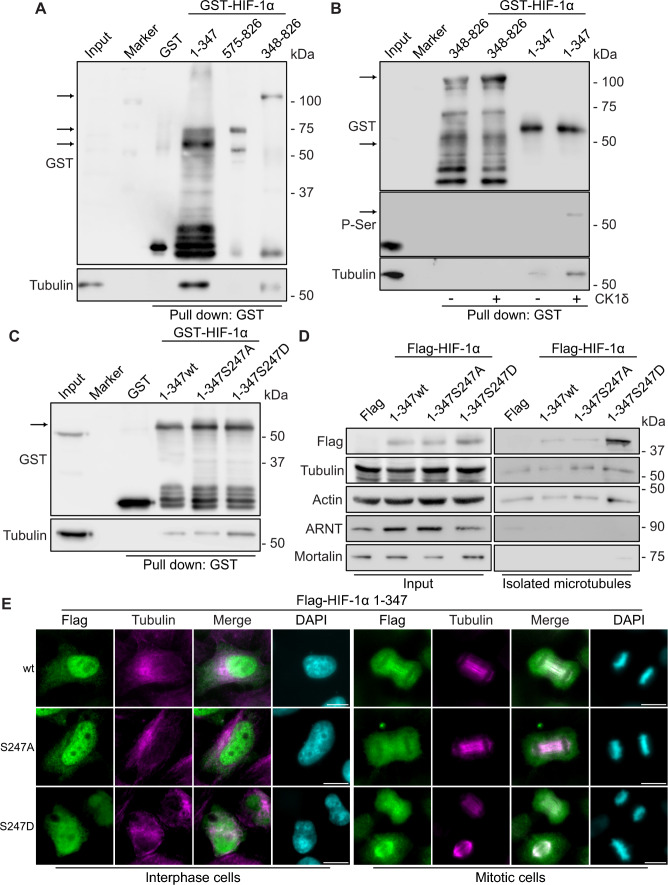
Increased association of HIF-1α N-terminal domain after its CK1δ phosphorylation with microtubule proteins. **A** Soluble HeLa protein extracts (Input) were mixed with GSH-agarose beads after immobilization of GST or different truncated forms of GST–HIF-1α (as indicated). Bound proteins (Pull-Down) were analyzed by immunoblotting using antibodies against GST or Tubulin proteins. **B** Soluble HeLa protein extracts (Input) were mixed with GSH-agarose beads after immobilization of GST or different N-terminal (1–347) or C-terminal (348–826) truncated forms of GST–HIF-1α that were previously in-vitro phosphorylated (or not) by purified CK1δ (as indicated). Bound proteins (Pull-Down) were analyzed by immunoblotting using antibodies against phosphoserine or GST and Tubulin proteins. **C** Soluble HeLa protein extracts (Input) were mixed with GSH-agarose beads after immobilization of GST or different N-terminal (1–347) phosphorylation mutant form of GST–HIF-1α (as indicated). Bound proteins (Pull-Down) were analyzed by immunoblotting using antibodies against GST or Tubulin proteins. **A**–**C** Arrows indicate the position of intact bacterially expressed purified proteins. **D** Soluble HeLa protein extracts (Input) or stabilized microtubule proteins from HeLa cells expressing either Flag or Flag-tagged N-terminal (1–347) phosphorylation mutant forms of HIF-1α (as indicated) were analyzed by immunoblotting using antibodies against Flag-epitope, Tubulin or Actin proteins. ARNT and Mortalin were used as microtubule purity controls. **E** Immunofluorescence microscopy images of HeLa *HIF1A*^−/−^ cells expressing either Flag or Flag-tagged N-terminal (1–347) phosphorylation mutant forms of HIF-1α (as indicated) using an antibody against tubulin to visualize microtubules. Nuclei were stained with DAPI (Scale bars: 10 μm)

To verify the interaction between HIF-1α and tubulin and to map the interaction site, pull-down assays with total HeLa cell extracts were performed using as baits recombinant GST-tagged parts of HIF-1α comprising amino acids 1–347 (bHLH and PAS domains), 575–826 (ETD and TAD domains), 348–826 (ODDD, ETD, and TAD domains) in wild-type and unmodified form. As shown in Fig. 5A, tubulin from the HeLa cell extracts bound strongly to the N-terminal part (amino acids 1–347) of HIF-1α, which contains the PAS domain and the CK1δ modification target, but its association with the C-terminal parts of HIF-1α was undetectable (for the 575–826 part) or very weak and not reproducibly detectable (for the 348–826 part; see also Sup. Fig. S2A for a repetition of the same experiment). Moreover, in-vitro phosphorylation of the HIF-1α fragments by recombinant CK1δ enhanced the binding of GST-HIF-1α 1–347 to tubulin but did not cause detectable association between GST-HIF-1α 348–826 and tubulin (Fig. 5B), pointing to the involvement of CK1δ and its Ser247 target in the regulation of the HIF-1α/tubulin interaction. Similar results were obtained in pull-down assays with GST-HIF-1α 1–347 fragments carrying mutations in the CK1δ phosphorylation site. As shown in Fig. 5C, the HIF-1α N-terminal domain harboring a phosphomimetic mutation at the CK1δ site (S247D) bound more strongly to tubulin than the corresponding (unmodified) wild-type or phosphodeficient S247A mutant form.

To further test the CK1δ-phosphorylation-dependent presence of HIF-1α on microtubules via its N-terminal domain, microtubules from extracts of HeLa cells expressing Flag-tagged forms of HIF-1α 1–347 were in-vitro stabilized with paclitaxel and recovered with ultracentrifugation [41]. As shown in Fig. 5D, recovery in the stabilized microtubule fraction was much stronger for the Flag-HIF-1α 1–347 fragment carrying the phosphomimetic S247D mutation than the corresponding wild-type or the phosphodeficient S247A mutant fragment. As a negative control, a nuclear (ARNT) or a mitochondrial (GRP75; mortalin) marker protein were virtually absent from the microtubule fractions (Fig. 5D). Immunofluorescence microscopy of HeLa *HIF1A*^−/−^ cells expressing the same Flag-HIF-1α 1–347 forms revealed two types of signals. In interphase cells, most of Flag-HIF-1α 1–347 was nuclear (Fig. 5E, left panels), probably due to the presence of a conserved nuclear localization sequence (NLS; aa 17–33) that is functional in truncated HIF-1α forms [25] or its import via importins 4/7 that recognize the HIF-1α N-terminal part [24]. However, the remaining cytoplasmic Flag-HIF-1α 1–347 S247D mutant clearly co-localized with tubulin, unlike the corresponding wild-type or phosphodeficient S247A mutant form (Fig. 5E, left panels). In the small population of cells undergoing mitosis, a certain pool of the Flag-HIF-1α 1–347 S247D mutant as well as the corresponding wild-type fragment co-localized strongly with mitotic spindle microtubules unlike the phosphodeficient S247A mutant, the co-localization of which with tubulin was barely observable, if at all present (Fig. 5E right panels; Sup. Fig. S2B). Thus, phosphorylation of HIF-1α by CK1δ inside its N-terminal PAS domain could potentially enhance its presence in microtubules during mitosis.

### CK1δ-mediated association of HIF-1α with mitotic microtubules ensures its symmetric distribution to daughter cell nuclei

**Fig. 6:**
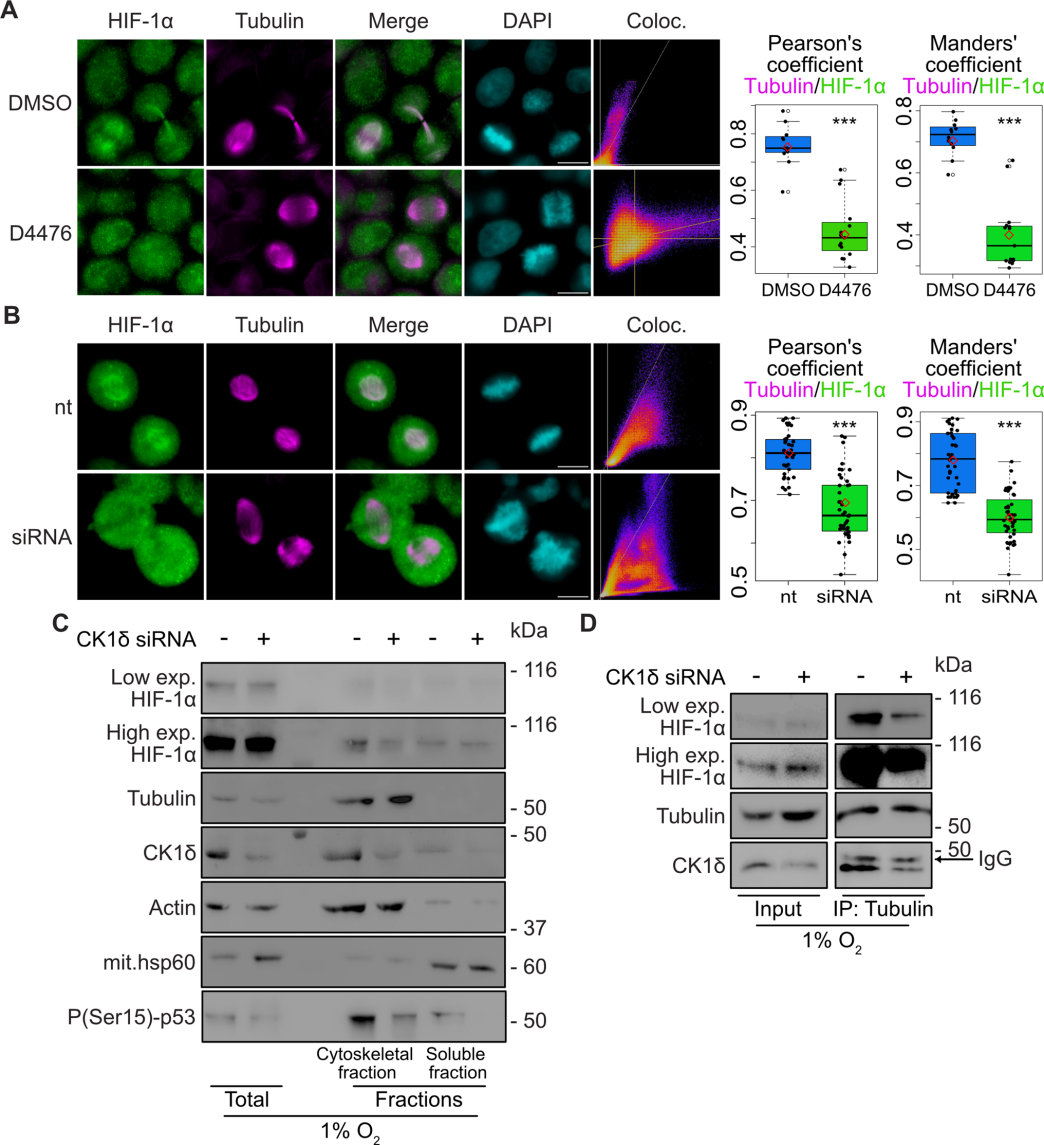
Co-localization of HIF-1α with microtubules during mitosis depends on CK1δ.** A** Immunofluorescence microscopy images of HeLa cells incubated at 1% O_2_ for 16 h in cells and treated (or not) with CK1δ inhibitor D4476 (as indicated) using antibodies against HIF-1α or tubulin. Nuclei were stained with DAPI (Scale bars: 10 μm). Scatterplots of pixel intensities of HIF-1α and tubulin signals are also shown. Boxplots show the Pearson’s (left panel), or Manders’ (right panel) overlap coefficients as measured in ~ 15 mitotic cells in each condition ± SEM (***P < 0.001; Filled circle: individual value, Empty circle: outlier, Solid line: median value, red diamond: mean value). **B** Immunofluorescence microscopy images of HeLa cells using antibodies against HIF-1α or tubulin. Cells were initially treated (or not) with CK1δ siRNA (as indicated) and incubated at 1% O_2_ for 16 h. During hypoxic treatment cells were synchronized for 6 h with nocodazole and were released for 1 h prior to fixation. Nuclei were stained with DAPI (Scale bars: 10 μm). Scatterplots of pixel intensities of HIF-1α and tubulin signals are also shown. Boxplots show the Pearson’s (left panel), or Manders’ (right panel) overlap coefficients as measured in ~ 35–40 mitotic cells in each condition ± SEM (***P < 0.001; Filled circle: individual value, Solid line: median value, red diamond: mean value). **C** Western Blot analysis of proteins recovered in soluble and microtubule-rich fractions (as indicated) of synchronized HeLa cells treated as in B, using antibodies against the indicated proteins. HIF-1α was detected after short (Low exp.) or long (High exp.) exposure time of the same membrane. Images are representative of two independent experiments. **D** Protein extracts (Input) or anti-Tubulin immunoprecipitated proteins (IP) of synchronized HeLa cells treated as in B, using antibodies against the indicated proteins. HIF-1α was detected after short (Low exp.) or long (High exp.) exposure time of the same membrane. Images are representative of two independent experiments. The arrow indicates the position of IgG heavy chain

To verify our findings with endogenous native HIF-1α, we, independently, treated HeLa cells grown under hypoxia (1% Ο_2_) with two CK1δ inhibitors D4476 or IC261. As expected, HIF-1α was nuclear in interphase cells, but in the subpopulation of mitotic cells, there was substantial co-localization between HIF-1α and spindle microtubules that was almost abolished in the presence of either D4476 or IC261 (Fig. 6A, Sup. Fig. S3A). To substantiate this result, CK1δ expression was silenced (Sup. Fig. S3B) in HeLa (Fig. 6B) or MCF7 (Sup. Fig. S3C) cells growing under hypoxia and subsequently synchronized with nocodazole, released from treatment, and analyzed by immunofluorescence microscopy. As above, there was substantial co-localization of HIF-1α with mitotic microtubules in control cells that was significantly reduced upon CK1δ silencing in both cell lines (Fig. 6B, Sup. Fig. S3C). These results were further verified by biochemical fractionation experiments in hypoxic and synchronized HeLa cells treated as above, which showed that recovery of endogenous HIF-1α in microtubule-enriched fractions was decreased when CK1δ expression was silenced (Fig. 6C). As a negative control, mitochondrial marker Hsp60 was mainly recovered in the soluble fraction. In the same experiment, the decrease in CK1δ activity because of its silencing was verified by the reduction in the phosphorylation of p53 Ser15, which is a known target of CK1δ (Fig. 6C; [33, 47]. In agreement with the fractionation results, HIF-1α as well as CK1δ were associated with immunoprecipitated tubulin from synchronized HeLa cells indicating the formation of a trimeric complex (Fig. 6D). Moreover, when cells were treated with CK1δ siRNA, the association of HIF-1α with tubulin was substantially decreased (Fig. 6D). Therefore, binding of endogenous HIF-1α to mitotic microtubules requires the presence and activity of CK1δ. This conclusion is further supported by co-localization experiments in mitotic cells showing that endogenous HIF-1α colocalized with CK1δ (Sup. Fig. S3D, upper panels), which, in turn, significantly colocalized with tubulin (Sup. Fig. S3D, lower panels).

**Fig. 7:**
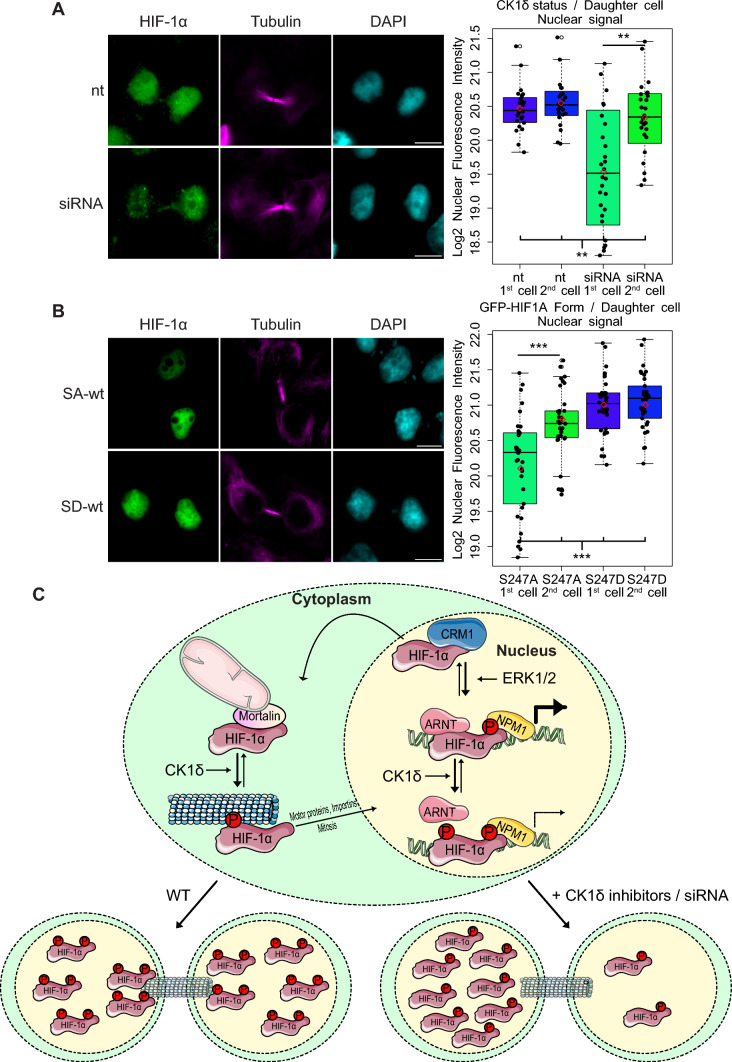
CK1δ-mediated phosphorylation of HIF-1α during mitosis facilitates efficient delivery of HIF-1α to daughter cell nuclei during cell division. **A** HeLa cells were initially treated (or not) with CK1δ siRNA (as indicated) and incubated at 1% O_2_ for 16 h. During hypoxic treatment cells were synchronized for 6 h with nocodazole and were released for 1 h prior to fixation and treated with antibodies against HIF-1α and tubulin. The images of newly formed nuclei at the end of cytokinesis (as indicated by tubulin staining) were processed for nuclear HIF-1α corrected fluorescence intensity signal after adaptive thresholding and transformed as log2 values. **B** HeLa *HIF1A*^−/−^ cells expressing GFP-HIF-1α SA-wt or SD-wt were incubated at 1% O_2_ for 16 h. During hypoxic treatment cells were synchronized for 6 h with nocodazole and were released for 1 h prior to fixation and treated with antibody against tubulin. The images of newly formed nuclei at the end of cytokinesis (as indicated by tubulin staining) were processed for nuclear HIF-1α corrected fluorescence intensity signal after adaptive thresholding and transformed as log2 values. Boxplots in **A** and **B** show the nuclear HIF-1α corrected fluorescence intensity (log2) in each daughter nuclei as measured in ~ 30–40 cells in each condition ± SEM (***P < 0.001; Filled circle: individual value, Empty circle: outlier, Solid line: median value, red diamond: mean value). **C** Schematic representation of HIF-1α combinatorial regulation by ERK1/2 and CK1δ kinases supports an equilibrium between nuclear and cytoplasmic HIF-1α functions. While inside the nucleus HIF-1α is phosphorylated by ERK1/2 to evade export by CRM1 and to stimulate gene expression by inducing association with NPM1 [18, 19]. Modification of nuclear HIF-1α by CK1δ weakens the interaction with ARNT and, subsequently, lowers HIF-1 activity [15]. Non-nuclear HIF-1α associates with mortalin on mitochondria to protect cells from apoptosis [20]. However, after its phosphorylation by CK1δ, non-nuclear HIF-1α preferentially associates with microtubules to potentiate its nuclear entry by motor proteins [35]. During cell division phosphorylation by CK1δ facilitates the symmetric distribution of HIF-1α to the two daughter nuclei. The figure depicts only the HIF-1α modifications mediated by ERK1/2 and CK1δ. For simplicity purposes, additional known HIF-1α modifications mediated by other kinases and their effects are not included in the model”

These findings raised the question of the functional significance of the HIF-1α/microtubule association, especially during mitosis. Association with the mitotic spindle, especially during anaphase and cytokinesis, may facilitate the symmetric distribution of a protein in the dividing cell and ensure its presence in both daughter cells in roughly equal amounts, which may be very important if the function of this protein is vital for the newly formed cells. To test if this is the case for HIF-1α, HeLa or MCF7 cells grown under hypoxia were synchronized by blocking mitosis at prometaphase with nocodazole for 6 h and observed with immunofluorescence microscopy 1 h after removing the nocodazole and releasing the arrest. As shown in representative images and their respective image analysis (Fig. 7A, Sup. Fig. S4), the endogenous HIF-1α signal was equal between nuclei in the two newly forming daughter cells (identified through their connection with the central spindle) in the control cells but differed significantly after silencing CK1δ expression in both cell lines, suggesting uneven distribution of HIF-1α in daughter nuclei when not modified by CK1δ. To confirm that the observed differences were connected to the modification status of HIF-1α and were not a nonspecific effect of CK1δ knock-down, we microscopically examined HeLa *HIF1A*^−/−^ cells that were expressing either the phospho-deficient GFP-HIF-1α SA-wt mutant or the phosphomimetic GFP-HIF-1α SD-wt mutant. Image analysis revealed that there was a significant difference in the distribution of GFP-HIF-1α SA-wt between daughter nuclei (Fig. 7B, upper panels), while, in contrast, the SD-wt form was evenly distributed between daughter nuclei (Fig. 7B, lower panels), corroborating the results with endogenous HIF-1α. Finally, to avoid the potentially toxic effects of nocodazole and to also perform a time-course experiment, we synchronized HeLa cells by blocking the cell cycle in the G2/M phase using the CDK1 inhibitor RO-3306. In addition, in this case (Sup. Fig. S5), daughter nuclei that had just formed presented similar signals for endogenous HIF-1α, while after CK1δ expression silencing, the signal was often weaker in one of the two daughter nuclei.

Taken together, our results suggest that CK1δ-mediated phosphorylation of HIF-1α and its subsequent association with spindle microtubules is necessary for the equal distribution and, possibly, nuclear import of HIF-1α between the newly forming daughter cells.

## Discussion

Cellular adaptation to an environment with limited oxygen supply requires induction of HIF-1, which activates many genes responsible for restraining mitochondrial oxygen consumption, reprogramming glucose and lipid metabolism, and ultimately promoting cell survival and preventing apoptotic cell death [48, 49]. Although the cellular oxygen-sensing machinery is centered around the stabilization of the HIF-1α subunit, it is not the sole determinant of the response to hypoxia, as there are numerous oxygen-independent processes that determine HIF-1α association with other regulatory proteins that ultimately fine-tune HIF-1 activity [50, 51]. A prevalent and efficient means to control these HIF-1α associations is through a variety of modifications, the most prominent being the phosphorylation [8]. We have previously reported two distinct phosphorylation events targeting HIF-1α with opposing functions; CK1δ-mediated modification targeting the N-terminal heterodimerization domain has an inhibitory effect, whereas ERK1/2 targeting the carboxy-terminal domain of HIF-1α stimulates HIF-1 activity [15, 16]. Here, by expressing a range of HIF-1α forms carrying combinations of CK1δ and ERK1/2 phosphosite mutations in a *HIF1A* knockout cell line, we provide evidence that the two phosphorylations have an impact on the versatility of HIF-1-mediated transcription and the HIF-1α non-transcriptional roles. Our results suggest that in the native HIF-1α form, both phosphorylation sites are partially modified, creating, thus, functionally distinct pools of HIF-1α. Concerning the transcriptionally inactive HIF-1α forms lacking modification by ERK1/2, our work indicates that they are not just cellular “waste”, but rather retain important non-genomic functions outside the nucleus. As we have previously shown, HIF-1α molecules that are not modified by ERK1/2 play an anti-apoptotic role after their translocation to mitochondria via mortalin by keeping BAX away from the outer mitochondrial membrane [20, 21]. In this work, we show that there is an additional level of control of non-nuclear HIF-1α mediated by CK1δ (Fig. 7C). Mutations mimicking CK1δ phosphorylation of non-nuclear HIF-1α resulted in a partial shift of HIF-1α localization from mitochondria to microtubules. The interaction of HIF-1α with tubulin was mediated by its N-terminal domain and was enhanced after its phosphorylation by CK1δ. Although the presence of HIF-1α on microtubules happened during interphase, it was much more evident during mitosis. Furthermore, the HIF-1α/microtubule association depended on the expression and activity of CK1δ and, ultimately, served the equal partitioning of HIF-1α to daughter nuclei at the end of cell division.

A drop in oxygen levels challenges cells for their survival. As mentioned, the presence of HIF-1α on mitochondria, which impairs BAX-mediated apoptosis, may represent an early pro-survival process [20, 21]. It could be argued that CK1δ-mediated phosphorylation of HIF-1α and its concomitant translocation onto microtubules could impair this mitochondrial HIF-1α function. However, our results have shown that this was not the case, as the non-nuclear HIF-1α form mimicking phosphorylation by CK1δ (SD-SA mutant form) largely retained its anti-apoptotic function. This may be attributed to the partial nature of the phenomenon, as a fraction of the HIF-1α SD-SA form amount remains bound to mitochondria, or to the dynamic interchange of HIF-1α between mitochondria and microtubules and/or to the association of mitochondria with microtubules that are known to facilitate their movement and permeability to energy metabolites [46].

Microtubules are dynamic structures and are influenced by oxygen concentrations due to p38-dependent phosphorylation of essential microtubule-associated proteins, albeit in a cell-specific manner [52]. Yet, the relationship between microtubules and hypoxia is bidirectional as their integrity is able to influence HIF-1α mRNA translation and protein accumulation [39, 53]. Moreover, in interphase cancer cells and cardiomyocytes, transport of HIF-1α to the nucleus was reported to be mediated by an intact microtubule network and via dynein activity [35, 54]. However, no specific signaling events that regulate the association of HIF-1α with the microtubular structure have been so far identified. Our results support the idea that non-nuclear HIF-1α can dynamically circulate between mitochondria and microtubules depending on its CK1δ phosphorylation status. This may be important for the transition between an early and a late response to hypoxia. As soon as oxygen levels drop, stabilization of unmodified HIF-1α and its localization to mitochondria may be able to prevent apoptosis caused by dysfunctional mitochondria starved of oxygen. As hypoxia persists, modification by CK1δ may trigger the association of HIF-1α with microtubules to facilitate the dynamic translocation of HIF-1α between cellular compartments and its import into the nucleus (Fig. 7C). In support of this notion, earlier studies have shown that CK1δ can also be localized on microtubules, targets both tubulins and microtubule-associated proteins and affects their dynamic instability [55, 56]. Notably, a significant fraction of overexpressed or endogenous HIF-1α displayed significant colocalization with the spindle microtubules and away from the condensed chromosomes. In accordance, CK1δ association with mitotic spindle components, which we have also observed in this work, has been previously found to be enhanced in synchronized cells, suggesting a role in shaping the mitotic microtubule interactome, along with other protein kinases such as Aurora and Plk [30]. In our case, both HIF-1α spindle localization and association with tubulin were impaired by inhibiting efficient phosphorylation of HIF-1α by CK1δ using point mutations, a CK1δ inhibitor, or CK1δ silencing. Although HIF-1α was nuclear at the end of cytokinesis, silencing of CK1δ or mutation of the CK1δ target site resulted in unequal partitioning of HIF-1α between the daughter nuclei. Such asymmetrical division of protein cargo during mitosis was previously observed for Smad1, β-catenin, or other polyubiquitinated proteins tagged for their proteasomal degradation [57]. So, it is possible that blocking CK1δ-mediated phosphorylation and the subsequent untimely release of HIF-1α from the spindle microtubules enhances its degradation and results in its asymmetric segregation.

The notion that the modification of HIF-1α from the same kinase, CK1δ, has two contrasting outcomes, on one hand facilitating its symmetric distribution during cell division and, on the other, weakening its interaction with ARNT inside the nucleus, seems paradoxical. However, as exemplified by our HIF-1α SD-SE mutant, HIF-1α retains a certain level of transcriptional activity even when modified by CK1δ provided that it is also modified by ERK1/2. Moreover, the CK1δ phosphorylation status of HIF-1α, once it is inside the nucleus, may be altered through the action of protein phosphatases. Protein phosphatases do indeed play a role in the regulation of HIF-1 activity [58, 59], but the phosphatases responsible for direct removal of the ERK1/2- or CK1δ-mediated modifications of HIF-1α remain unidentified.

The relation between cell cycle and HIF-1α is bidirectional, as hypoxia can cause the arrest of the cell cycle to reduce energy-demanding proliferative functions [60]. HIF-1α is directly implicated in this process both transcriptionally and in a non-genomic fashion by interfering in DNA replication [22, 61]. On the other hand, cell cycle regulators such as CDK1 and CDK2 directly target HIF-1α and affect its function. CDK1 phosphorylates HIF-1α at Ser668 and promotes HIF-1α stabilization during G2/M phases [14]. CDK2 has a dual effect on HIF-1α as it promotes HIF-1α degradation during G1 to S transition (a process counteracted by CDK1/CyclinB), whereas it upregulates HIF-1α-mediated gene expression at S/G2 phases [13]. Moreover, it was recently reported that under normoxia, HIF-1α is transiently expressed during the G1 phase in an AMP-activated protein kinase (AMPK)-dependent manner and in cancer cells grown under nutritional stress contributing, thus, to their survival [62]. In line with these studies, our results suggest that various distinct phosphorylation events finely tune not only HIF-1α stabilization but also its subcellular distribution and activity during the cell cycle.

Given the importance of HIF-1α for cancer development and its establishment as a promising therapeutic target, our work identified a previously unknown oxygen-independent mechanism that involves CK1δ and the microtubule cytoskeleton and regulates HIF-1α localization and nuclear accumulation at a critical step of the cell cycle such as cell division. As all the components of this tripartite system can be targeted [30, 35, 63], understanding the details of their association could have important implications for controlling cancer progression.

### Supplementary Information

Below is the link to the electronic supplementary material.

Supplementary file1 (XLSX 13 KB)

Supplementary file2 (PDF 14643 KB)

Supplementary file3 (PDF 65 KB)

## Data Availability

The proteomic data discussed in this publication are shown in the supplementary file Sup_File_S1.

## Abbreviations

AMPK: AMP-activated protein kinase
ARNT: Aryl hydrocarbon receptor nuclear translocator
BAX: Bcl-2-associated X protein
BSA: Bovine serum albumin
CDK: Cyclin-dependent kinase
CK1δ: Casein Kinase 1δ
CRM1: Chromosomal region maintenance 1
DAPI: 4,6-Diamidino-2-phenylindole
DMEM: Dulbecco's Modified Eagle's Medium
DMSO: Dimethyl-sulfoxide
DTT: Dithiothreitol
ERK1/2: Extracellular signal-regulated kinase 1/2
ΕTD: ERK-Targeted domain
FBS: Fetal Bovine Serum
GFP: Green fluorescent protein
GSH: Glutathione
GST: Glutathione S-transferase
HIF-1: Hypoxia-inducible factor-1
ΗΚΙΙ: Hexokinase II
HRE: Hypoxia Response Elements
MAPs: Microtubule-associated proteins)
NPM1: Nucleophosmin 1
PHD: Prolyl hydroxylase domain
VDAC1: Voltage dependent anion channel 1
VHL: Von Hippel Lindau
