# Supplementary material for: Combinatorial regulation by ERK1/2 and CK1δ protein kinases leads to HIF-1α association with microtubules and facilitates its symmetrical distribution during mitosis
Source: Cell Mol Life Sci. 2024 Feb 1;81(1):72. doi: 10.1007/s00018-024-05120-7 (PMC10834586; doi:10.1007/s00018-024-05120-7)
Supplement: Supplementary file 2 — Supplementary file2 (PDF 14643 KB) [file 18_2024_5120_MOESM2_ESM.pdf]

**Supplemental Material**

**Combinatorial regulation by ERK1/2 and CK1δ protein kinases leads to HIF-1α association with microtubules and facilitates its symmetrical distribution during mitosis.**

Christina Arseni[1], Martina Samiotaki[2], George Panayotou[2], George Simos[1*], Ilias Mylonis[1*]

**Supplemental material**

**Figure S1** relates to **Figures 1 and 4** and contains additional immunoblotting analysis (relevant to **Fig.1**) or caspase 3/7 activity experiments (relevant to **Fig.4**) to measure apoptotic potential of cells expressing GFP-HIF-1α mutants in the presence of etoposide.

**Figure S2** relates to **Figure 5** and contains Western Blot analysis of an independent pull-down assay (relevant to **Fig.5A)** and additional immunofluorescence analysis (relevant to **Fig.5E)**.

**Figure S3** relates to **Figure 6** and contains additional immunofluorescence and Western Blot analysis.

**Figure S4** relates to **Figure 7** and contains immunofluorescence experiments in an additional cell line (MCF7).

**Figure S5** relates to **Figures 6&7** and contains additional immunofluorescence images acquired at different time points after the release of synchronized HeLa cells.

**Sup_File_S1** relates to **Figure 4** and contains the lists of GFP-HIF-1α wt-SA or GFP-HIF-1α SD-SA interacting partners identified by MS and their analysis.

**Tables S1-S5** contain lists of reagents, kits, and chemicals used in this study and relate to **Material and Methods, and Results sections.**

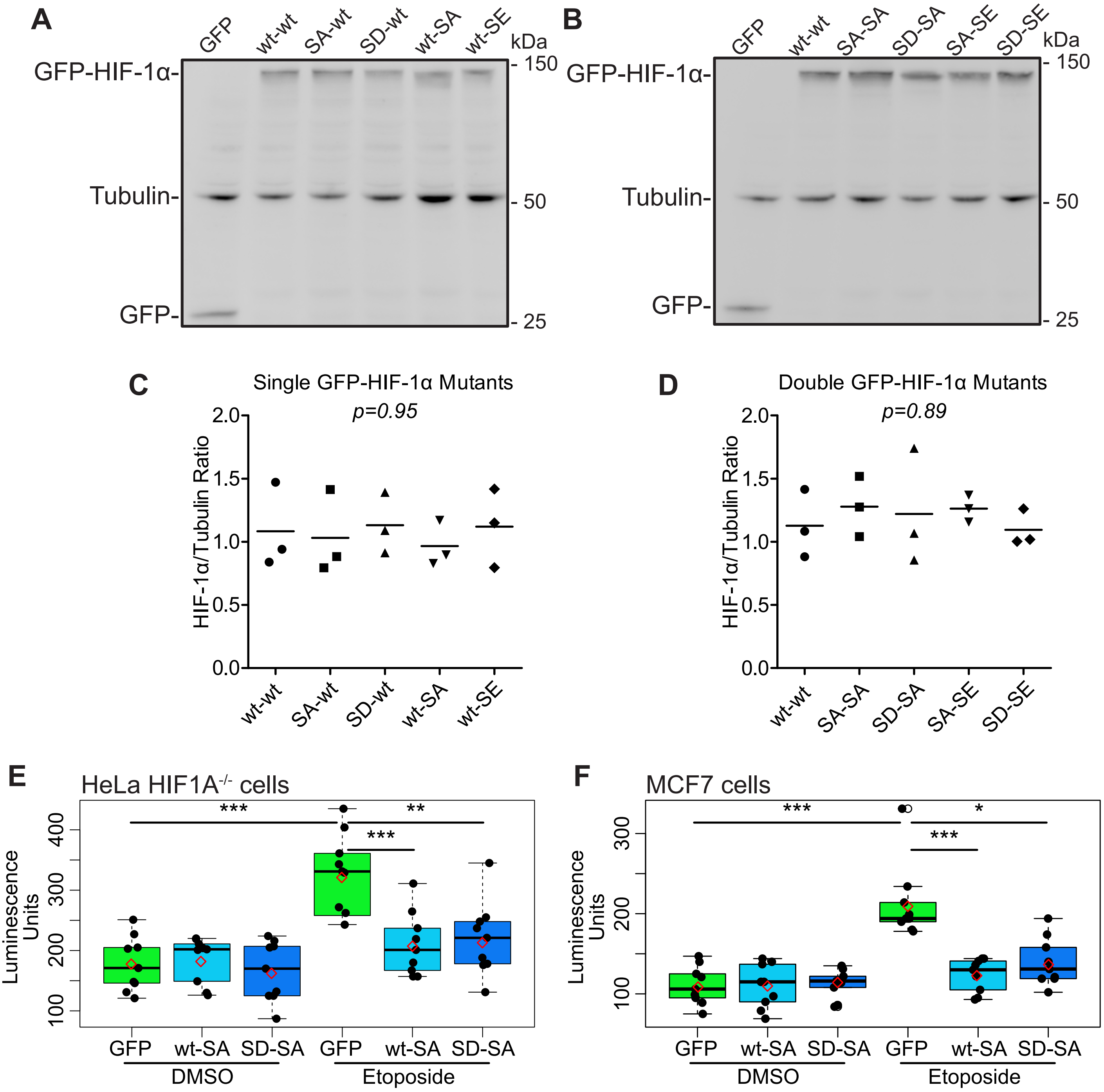


SUP. FIGURE S1

**Sup. Figure S1**

**A&B.** Western Blot analysis of GFP-tagged HIF-1α phosphorylation mutant forms carrying single (**A**) or double (**B**) mutations expressed in HeLa *HIF1A*$^{-/-}$ cells under hypoxia (1% $O_2$) that were initially incubated with an antibody against HIF-1α (as shown in **Fig. 2B**) and the reblotted using antibodies against GFP and Tubulin (as indicated).

**C&D.** Quantitative analysis of immunoblots from three independent experiments (as represented in **Fig. 2B**) depicting the HIF-1α / Tubulin ratio of GFP-tagged HIF-1α phosphorylation mutant forms carrying single (**C**) or double (**D**) mutations (as indicated). Shown are the ratio values from three independent experiments, including mean (lines) and P values.

**E&F.** Determination of caspase 3/7 activity in HeLa *HIF1A*$^{-/-}$ (**E**) or MCF7 (**F**) cells expressing GFP alone, GFP-HIF-1α-S641/3A or GFP-HIF-1α-SDSA and treated 24 h post-transfection with either DMSO (control) or 75 µM of etoposide for 4h in normoxia. Results are expressed as mean (red diamonds) +/-SEM (n=9; **$p < 0.01$; ***$p<0.001$; Filled circle: individual value, Empty circle: outlier, Solid line: median value).

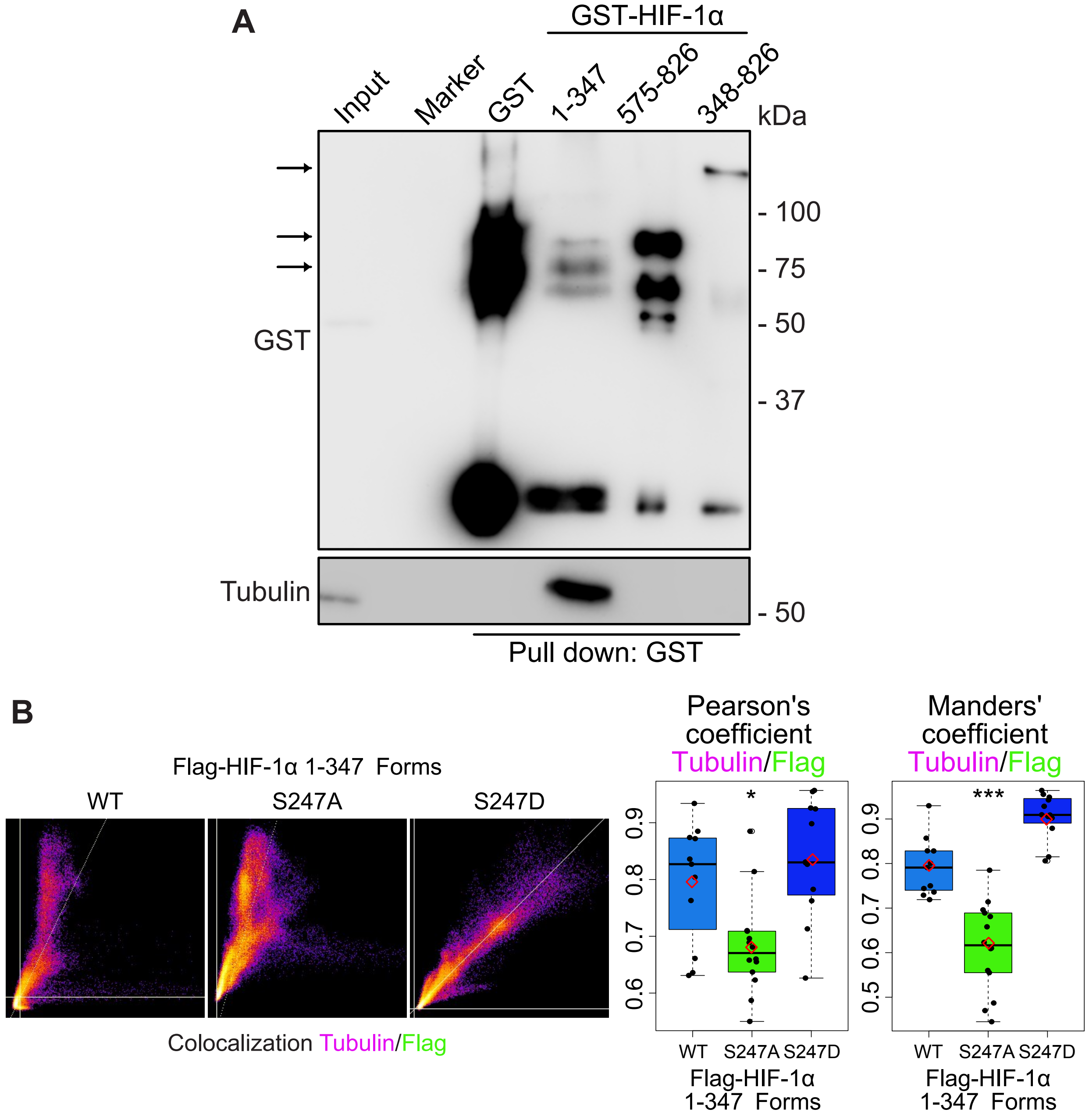


SUP. FIGURE S2

**Sup. Figure S2**

Localization of HIF-1α to microtubules.

**A.** An independent pull-down assay performed as the one shown in **Fig. 5A**. Soluble HeLa protein extracts (Input) were mixed with GSH-agarose beads after immobilization of GST or different truncated forms of GST–HIF-1α (as indicated). Bound proteins (Pull-Down) were analyzed by immunoblotting using antibodies against GST or Tubulin proteins.

**B.** Analysis of immunofluorescence microscopy images shown in **Fig. 3E**. Scatterplots of pixel intensities of Flag and tubulin signals for the different Flag-HIF-1α (1-347) phosphorylation mutant forms (as indicated). Boxplots show the Pearson's (left panel), or Manders' (right panel) overlap coefficients as measured in ~12 cells in each condition +/-SEM (*$p < 0.05$, (***$p < 0.001$; Filled circle: individual value, Solid line: median value, red diamond: mean value).

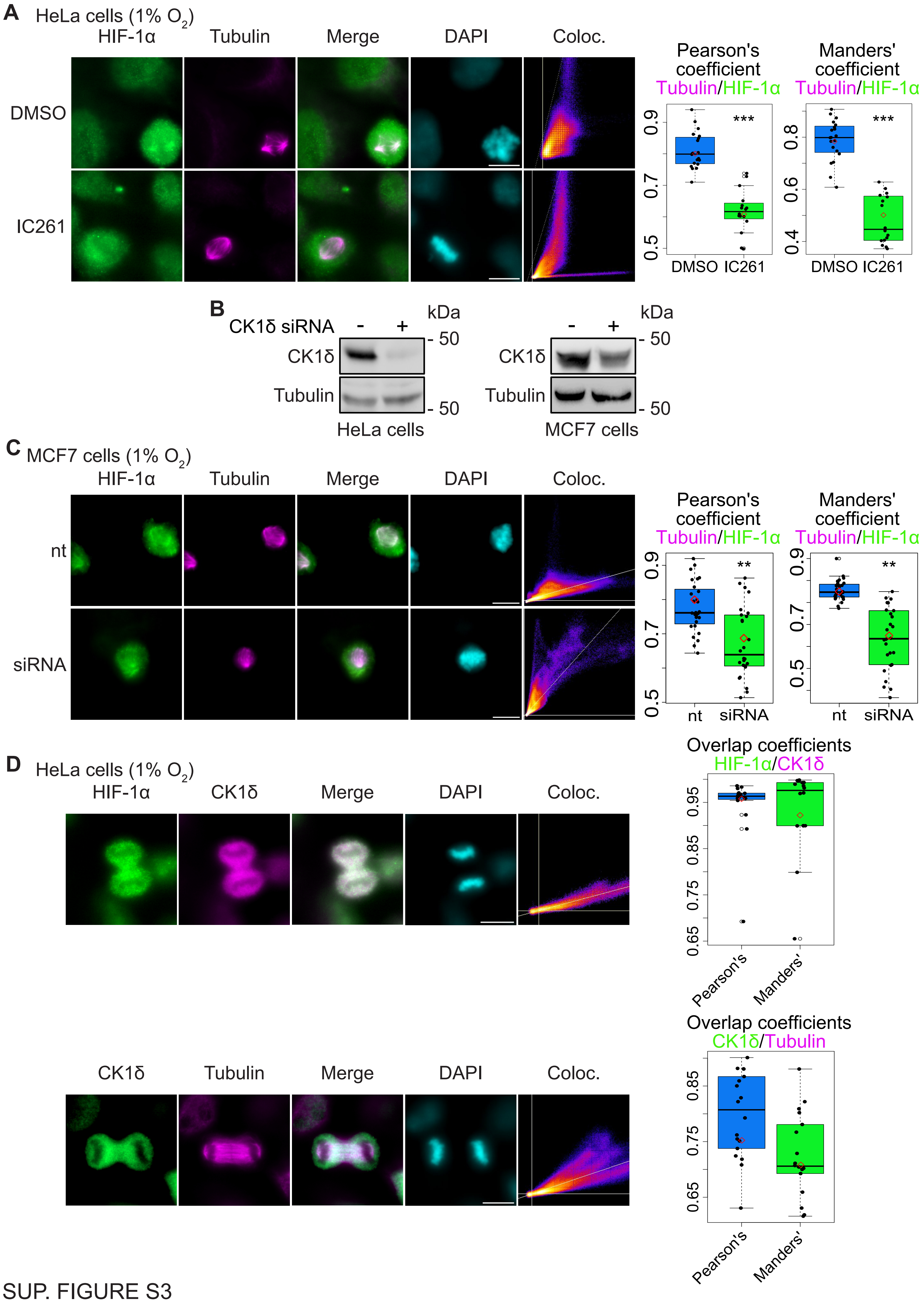


SUP. FIGURE S3

**Sup. Figure S3**

Localization of HIF-1α to microtubules during mitosis depends on CK1δ.

**A.** Immunofluorescence microscopy images of HeLa cells incubated at 1% $O_2$ for 16 h in cells and treated (or not) with CK1δ inhibitor IC261 (as indicated) using antibodies against HIF-1α or tubulin. Nuclei were stained with DAPI (Scale bars: 10 µm). Scatterplots of pixel intensities of HIF-1α and tubulin signals are also shown. Boxplots show the Pearson's (left panel), or Manders' (right panel) overlap coefficients as measured in ~20 mitotic cells in each condition +/-SEM (***$P < 0.001$; Filled circle: individual value, Empty circle: outlier, Solid line: median value, red diamond: mean value).

**B.** Western Blot analysis of HeLa or MCF7 cells (as indicated) shown in **Fig. 6B** or **Sup. Figs. S3C&S4** after CK1δ siRNA treatment, using antibodies against the indicated proteins.

**C.** Immunofluorescence microscopy images of MCF7 cells initially treated (or not) with CK1δ siRNA (as indicated) and incubated at 1% $O_2$ for 16 h. During hypoxic treatment cells were synchronized for 6h with nocodazole and were released for 1h prior to fixation and treated with antibodies against HIF-1α and tubulin. Nuclei were stained with DAPI (Scale bars: 10 µm). Scatterplots of pixel intensities of HIF-1α and tubulin signals are also shown. Boxplots show the Pearson's (left panel), or Manders' (right panel) overlap coefficients as measured in ~25 cells in each condition +/-SEM (**$p< 0.01$; Filled circle: individual value, Solid line: median value, red diamond: mean value).

**D.** Immunofluorescence microscopy images of HeLa cells incubated at 1% $O_2$ for 16 h. After fixation cells were stained using antibodies against HIF-1α and CK1δ (upper panels) or CK1δ and tubulin (lower panels). Nuclei were stained with DAPI (Scale bars: 10 µm). Scatterplots of pixel intensities of HIF-1α and CK1δ (upper panels) or CK1δ and tubulin (lower panels) signals are also shown. Boxplots show the Pearson's or Manders' (as indicated) overlap coefficients as measured in ~18 mitotic cells in each condition +/-SEM (***$P < 0.001$; Filled circle: individual value, Empty circle: outlier, Solid line: median value, red diamond: mean value).

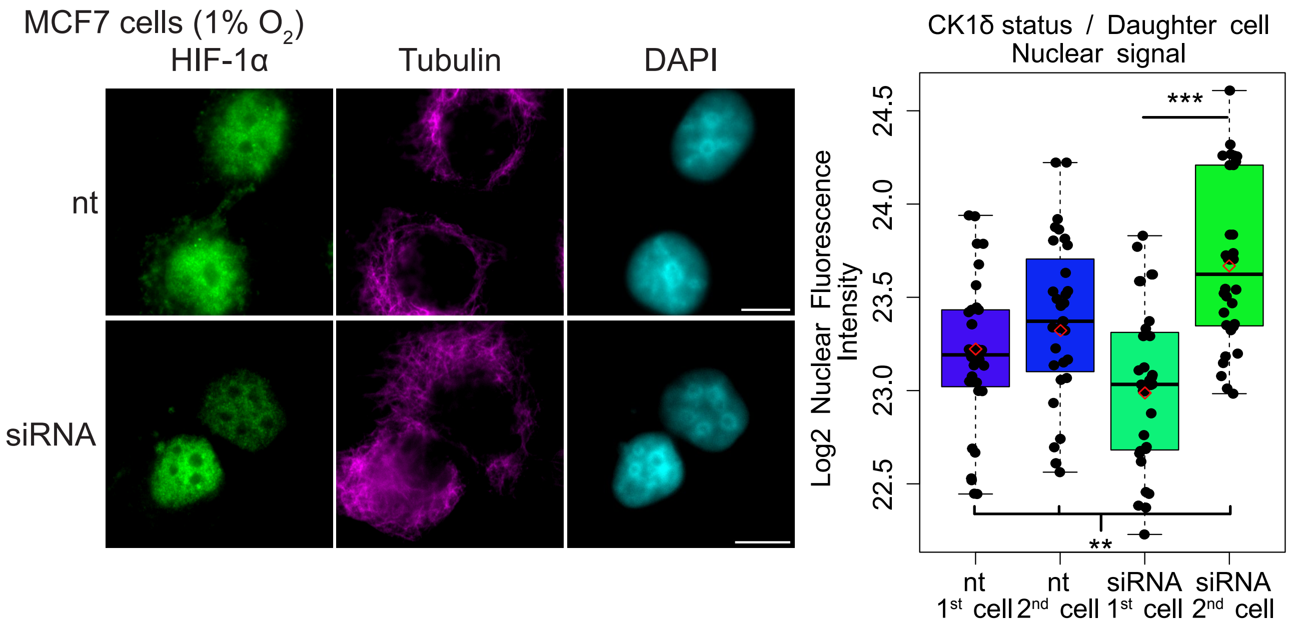


SUP. FIGURE S4

**Sup. Figure S4**

Localization of HIF-1α to microtubules during mitosis facilitates efficient delivery of HIF-1α to daughter cell nuclei during cell division.

Immunofluorescence microscopy images of synchronized MCF7 cells treated (or not) with CK1δ siRNA (as indicated) and incubated at 1% $O_2$ for 16 h. During hypoxic treatment cells were synchronized for 6h with nocodazole and were released for 1h prior to fixation and treated with antibodies against HIF-1α and tubulin. Determination of HIF-1α nuclear signal was performed as described in **Fig. 7**. Boxplots show the nuclear HIF-1α corrected fluorescence intensity (log2) in each daughter nuclei as measured in ~25-30 cells in each condition +/-SEM (**$p < 0.01$; ***$p$<0.001; Filled circle: individual value, Empty circle: outlier, Solid line: median value, red diamond: mean value).

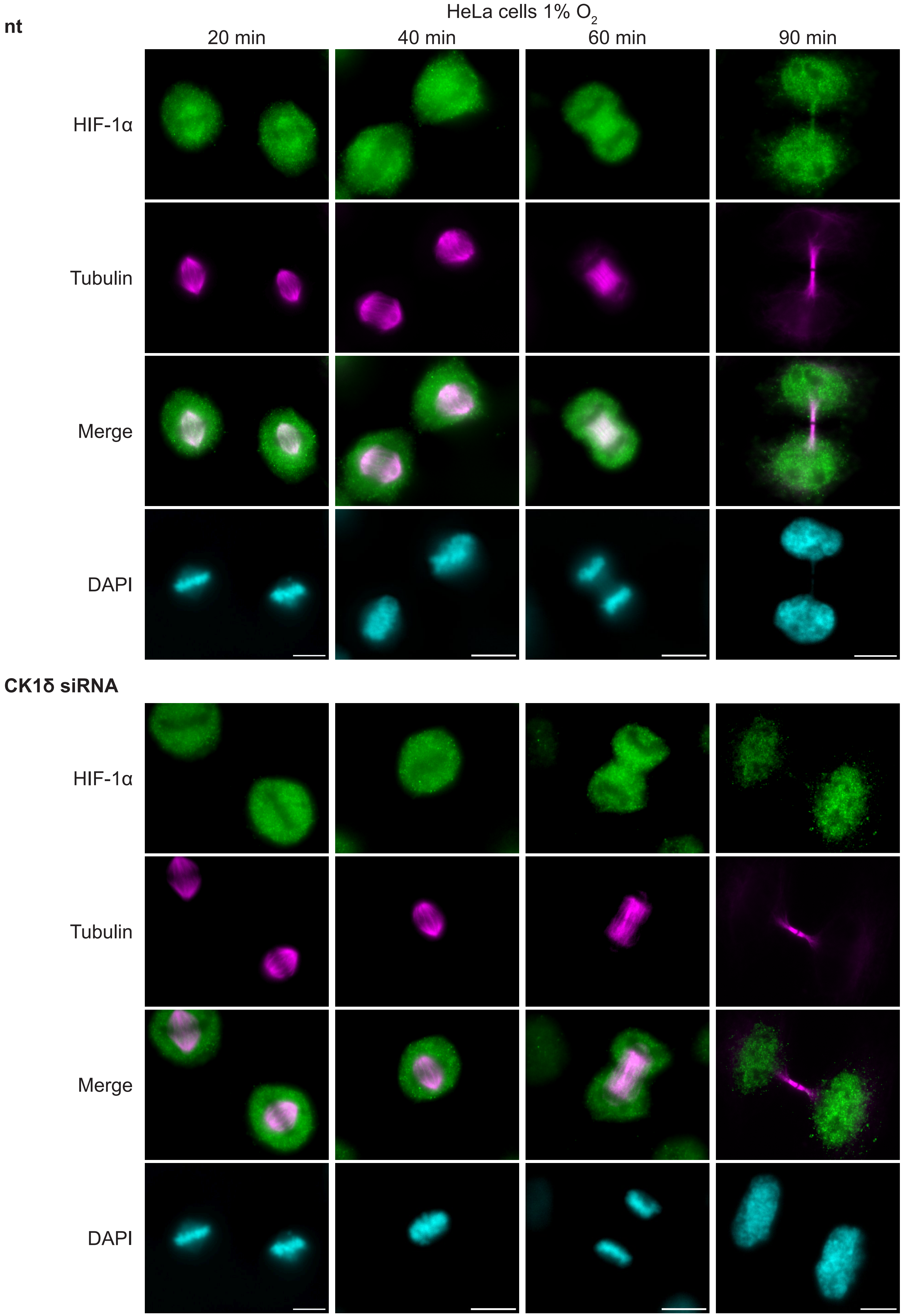


SUP. FIGURE S5

**Sup. Figure S5**

Localization of HIF-1α to microtubules during mitosis facilitates efficient delivery of HIF-1α to daughter cell nuclei.

Immunofluorescence microscopy images of HeLa cells treated (or not) with CK1δ siRNA (as indicated) and incubated at 1% $O_2$ for 20h in the presence of 5 µM RO-3306. Cells were released at the indicated times prior to fixation and treated with antibodies against HIF-1α and tubulin. Nuclei were stained with DAPI (Scale bars: 10 µm).
