# Supplementary material for: Combinatorial regulation by ERK1/2 and CK1δ protein kinases leads to HIF-1α association with microtubules and facilitates its symmetrical distribution during mitosis
Source: Cell Mol Life Sci. 2024 Feb 1;81(1):72. doi: 10.1007/s00018-024-05120-7 (PMC10834586; doi:10.1007/s00018-024-05120-7)
Supplement: Supplementary file 3 — Supplementary file3 (PDF 65 KB) [file 18_2024_5120_MOESM3_ESM.pdf]

Table S1. List of Primers used in this study.

Primers for cloning and mutagenesis

Primer	Sequence (5'-3')
HIF-N-B (Cloning)	5'-TTT TTG GAT CCC GAT TCA CC**A TG**G AGG GC- 3'
H347R (Cloning)	5'-TTT TTG GAT CC**T CA**C TGA ATA ATA CCA CTC ACA ACG- 3'
S247D Forward	5'GACTTTCCTCAGTCGACACGACCTGGATATGAAATTTTC-3'
S247D Reverse	5'-GAAAATTTCATATCCAGGTCGTGTTCGAC-3'
S247A Forward	5'GACTTTCCTCAGTCGAGACGCCCTGGATATGAAATTTTC-3'
S247A Reverse	5'-GAAAATTTCATATCCAGGGAGGAAAGTC-3'

Primers for RT-PCR

Primer	Sequence (5'-3')
P4HA1	F 5'-AGGGGTTGCTGTGGATTACC-3'<br>R 5'-GTCATGTACTGTAGCTCGGC-3'
HIF1A	F 5‘ GGATTAGTTCTGAACGTCGA 3‘<br>R 5‘ AGTTAGGGTACACTTC 3‘
ACTIN	F 5' CCAACCGCGAGAAGATGA 3'<br>R 5' CCAGAGGCGTACAGGGATAG 3'

Table S2. List of transfection agents and Kits

QuikChange II mutagenesis kit	Agilent, Santa Clara CA, USA
Caspase-Glo® 3/7	Promega, Madison, WI, USA
JetPRIME® Polyplus	Polyplus, Strasbourg, France
VIROMER®BLUE	Biontech, Germany
NucleoZOL	MACHEREY-NAGEL, Germany
High-Capacity Reverse Transcription kit	Applied Biosystems, Foster City, CA, USA
SYBR™ Select Master Mix	Applied Biosystems, Foster City, CA, USA
HIF1A CRISPR/Cas9 Double Nickase	Santa Cruz Biotechnology, Dallas, TX, USA
Dual-Luciferase Reporter Assay System	Promega, Madison, WI, USA

Table S3. List of Chemicals and reagents

Chemical/reagent	Reference
Recombinant CK1δ	P6030, New England Biolabs, USA
Etoposide	E1383, Sigma-Aldrich, Inc, USA
D4476	13305, Cayman, USA
DAPI	D9542, Sigma-Aldrich, Inc, USA
Mitotracker	M46751, Invitrogen, Life Technologies, Carlsbad, CA)
Nocodazole	*M1404,* Sigma-Aldrich, Inc, USA
Puromycin	A1113802, Gibco, USA
Taxol	T7191, Sigma-Aldrich, Inc, USA
RO-3306	217699, Sigma-Aldrich, Inc, USA
DMSO	A3672, AppliChem,Illinois, USA
IC261	400090, Sigma-Aldrich, Inc, USA

Table S4. List of non-target and specific siRNAs

siRNA	Sequence (5'-3')	Reference
AllStars Non-target siRNA	Proprietary	1027280, Qiagen, USA
CK1δ siRNA	Proprietary (Pool of 3 target-specific 19-25 nt siRNAs)	29910, Santa Cruz Biotechnology, Dallas, USA

Table S5. List of antibodies used in this study.

Primary antibodies

Antibody	Reference	Working dilution
Mouse Anti- β- Actin	3700S, Cell Signaling, USA	WB 1:5000
Mouse Anti- α -Tubulin	3873S, Cell Signaling, USA	WB 1:10000<br>IF 1:2000
Rabbit Anti- β -Tubulin	2128S, Cell Signaling, USA	IF 1:100
Rabbit Anti-GFP	2956S, Cell Signaling, USA	WB 1:1000
Goat Anti-GFP	AB0020-200, Sicgen, Portugal	WB 1:2000<br>IP 1µg per 1mg protein
Rabbit Anti-HSP60	4870, Cell Signaling, USA	WB 1:1000
Rabbit Anti-CK1δ	12417, Cell Signaling, USA	WB 1:1000
Rabbit Anti-CK1δ	14388-1-AP, Proteintech, Germany	IF 1:200
Rabbit Anti-GRP75	13967, Santa Cruz Biotechnology, Dallas, USA	WB 1:1000
Mouse Anti-GST(B-14)	138, Santa Cruz Biotechnology, Dallas, USA	WB 1:1000
Mouse Anti-Flag	1804, Sigma-Aldrich, Inc, USA	WB 1:3000<br>IF 1:1000
Mouse Anti-HIF-1α	610959, BD Biosciences, USA	WB 1:500<br>IF 1:250
Mouse Anti-P-Ser	612547, BD Biosciences, USA	WB 1:4000
Rabbit Anti-Phospho-p53 (Ser15)	9284, Cell Signaling, USA	WB 1:1000
Mouse Anti-ARNT	611079, BD Biosciences, USA	WB 1:500

Secondary antibodies

Antibody	Reference	Working dilution
Goat Anti-Rabbit HRP	AP187P, Sigma-Aldrich, Inc, USA	WB 1:5000
Horse Anti-Mouse HRP	7076, Cell Signaling, USA	WB 1:5000
Mouse Anti-Goat HRP	Sc-2354, Santa Cruz Biotechnology, Dallas, USA	WB 1:2000
Donkey Anti-Rabbit IgG (Cy3)	711-165-152, Jackson ImmunoResearch, UK	IF 1:500
Anti-mouse IgG Fab2 488	4408S, Cell Signaling, USA	IF 1:500
Anti-rabbit IgG Fab2 488	4412S, Cell Signaling, USA	IF 1:500
CyTM3-Goat Anti-Mouse IgG (H+L) Conjugate (ZyMaxTM Grade)	81-6515, Invitrogen, USA	IF 1:500
Goat Anti-Rabbit IgG (H+L) alexa fluor 647	A27040, Invitrogen, USA	IF 1:500
